# Hyper-phosphorylation of Rb S249 together with CDK5R2/p39 overexpression are associated with impaired cell adhesion and epithelial-to-mesenchymal transition: Implications as a potential lung cancer grading and staging biomarker

**DOI:** 10.1371/journal.pone.0207483

**Published:** 2018-11-19

**Authors:** Jaileene Pérez-Morales, Darielys Mejías-Morales, Stephanie Rivera-Rivera, Jonathan González-Flores, Mónica González-Loperena, Fernando Y. Cordero-Báez, Wilfredo M. Pedreira-García, Camille Chardón-Colón, Jennifer Cabán-Rivera, W. Douglas Cress, Edna R. Gordian, Teresita Muñoz-Antonia, Mauricio Cabrera-Ríos, Angel Isidro, Domenico Coppola, Marilin Rosa, Theresa A. Boyle, Victoria Izumi, John M. Koomen, Pedro G. Santiago-Cardona

**Affiliations:** 1 Biochemistry and Cancer Biology Divisions, Basic Science Department, Ponce Health Sciences University-Ponce Research Institute, Ponce, Puerto Rico; 2 Molecular Oncology and Thoracic Oncology Departments, H. Lee Moffitt Cancer Center and Research Institute, Tampa, Florida, United States of America; 3 Department of Industrial Engineering, University of Puerto Rico at Mayagüez, Mayagüez, Puerto Rico; 4 Physiology Division, Basic Science Department, Ponce Health Sciences University-Ponce Research Institute, Ponce, Puerto Rico; 5 Anatomic Pathology, Moffitt Cancer Center and Research Institute, Tampa, Florida, United States of America; 6 Proteomics, H. Lee Moffitt Cancer Center and Research Institute, Tampa, Florida, United States of America; University of South Alabama Mitchell Cancer Institute, UNITED STATES

## Abstract

Prediction of lung cancer metastasis relies on post-resection assessment of tumor histology, which is a severe limitation since only a minority of lung cancer patients are diagnosed with resectable disease. Therefore, characterization of metastasis-predicting biomarkers in pre-resection small biopsy specimens is urgently needed. Here we report a biomarker consisting of the phosphorylation of the retinoblastoma protein (Rb) on serine 249 combined with elevated p39 expression. This biomarker correlates with epithelial-to-mesenchymal transition traits in non-small cell lung carcinoma (NSCLC) cells. Immunohistochemistry staining of NSCLC tumor microarrays showed that strong phospho-Rb S249 staining positively correlated with tumor grade specifically in the squamous cell carcinoma (SCC) subtype. Strong immunoreactivity for p39 positively correlated with tumor stage, lymph node invasion, and distant metastases, also in SCC. Linear regression analyses showed that the combined scoring for phospho-Rb S249, p39 and E-cadherin in SCC is even more accurate at predicting tumor staging, relative to each score individually. We propose that combined immunohistochemistry staining of NSCLC samples for Rb phosphorylation on S249, p39, and E-cadherin protein expression could aid in the assessment of tumor staging and metastatic potential when tested in small primary tumor biopsies. The intense staining for phospho-Rb S249 that we observed in high grade SCC could also aid in the precise sub-classification of poorly differentiated SCCs.

## Introduction

The retinoblastoma protein (Rb) is one of the most important tumor suppressors, as illustrated by the fact that either Rb itself or some of its pathway components is the target of oncogenic driver mutations in most, if not all, human cancers [1–8]. Rb has been canonically characterized as a cell cycle regulator [2,7], but we and others have characterized a novel non-traditional Rb function in the induction of cell-to-cell and cell-to-substrate adhesion [9–16]. We showed that Rb deletion abrogates cellular adhesion by preventing the formation of adherens junctions and by affecting the transcriptional profile of several cadherins and integrins [12–15].

Given that Rb’s function is regulated by phosphorylation, and that Rb inactivation by hyper-phosphorylation is a frequent occurrence in human cancers [2,7,17–19] and in light of Rb’s role in cell adhesion, we postulated that there is a specific Rb phosphorylation signature that abrogates Rb´s capacity to promote cell adhesion, and that such a phosphorylation signature could be a clinically informative biomarker for establishing metastatic potential based on a biopsy of a primary tumor. To investigate this, we conducted a liquid chromatography-tandem mass spectroscopy-based full-length phosphorylation mapping of Rb purified from two non-small cell lung carcinomas (NSCLC) cell lines; H520 cells, which are poorly adhesive and exhibit traits of epithelial-to-mesenchymal (EMT) transition, and H1666 cells, which express epithelial but not EMT-related markers, and have cell-to-cell adhesion. We found that in H520 cells Rb is hyper-phosphorylated on residues serine 249 (S249) and threonine 821 (T821) relative to H1666 cells. In H520 cells Rb hyper-phosphorylation in S249 and T821 coexists with lack of adherens junctions, low E-cadherin levels, increased N-cadherin and vimentin levels, diminished integrin α5 expression, activation of Focal Adhesion Kinase (FAK), diminished cell-to-cell contacts, and p39 overexpression. We thus hypothesized that Rb hyper-phosphorylation in S249 and T821 together with p39 over-expression could serve as a clinically informative biomarker for tumor stage, grade, and metastatic potential in pre-resection small NSCLC biopsy samples. Consistent with our hypothesis, we found that a strong phospho-Rb S249 staining positively correlated with tumor grade exclusively in squamous cell carcinomas (SCC) and not in adenocarcinomas (AC), meaning that most of the SCC tumors with higher grades also had high scores for phospho-Rb S249 positive cells. This indicates that Rb S249 phosphorylation persists in poorly differentiated SCCs distinguishing them from poorly differentiated AC. Regarding p39 immunostaining, we found the most significant correlations also in SCC, where p39 staining positively correlated with tumor staging, lymph node invasion, and distant metastases. Linear regression analyses showed that the combined score for phospho-Rb S249 and p39 is even more clinically informative than either score alone, predicting tumor stage with good precision. Based on our data, we propose that pathologists should perform combined immunohistochemistry staining of lung tumor samples for Rb S249 phosphorylation and for p39 protein expression to aid in the clinical assessment of SCC tumor staging, histological NSCLC subtyping and metastatic potential in those patients who are not amenable to tumor resection and for whom treatment must be informed predominantly by evaluations of small tumor biopsies.

## Materials and methods

### Cell Cultures and cell line authentication and genotyping

NSCLC cell lines were obtained from the H. Lee Moffitt Lung Cancer Center of Excellence Cell Line Core and maintained in RPMI medium supplemented with 10% heat-inactivated fetal bovine serum and 1% Streptomycin/Penicillin. The cell lines used were the following: NCI-H1666 (ATCC CRL-5885, human adenocarcinoma), NCI-H520 (ATCC HTB-182, human, squamous cell carcinoma), NCI-H1650 (ATCC CRL-5883, human adenocarcinoma), NCI-1975 (ATCC CRL-5908, human adenocarcinoma), NCI-H596 (ATCC HTB-178, human adenosquamous carcinoma), and NCI-H60 (ATCC CRL-5821, human small cell lung carcinoma). Cell lines were cultured in 100 mm plates and kept in a humidified water-jacketed incubator at 37°C and 5% CO_2_ and passaged every 5–7 days to maintain a confluence of approximately 80%. Cell lines tested negative for mycoplasma contamination and were authenticated by short tandem repeat (STR) analysis of loci TH01, TPOX, vWA, CSF1PO, D16S539, D7S820, D13S317 and D5S818. Genotypes of both cell lines were based on the Cell Line Encyclopedia as previously described [20].

### Immunofluorescence

Immunofluorescence was performed following standard methods. Briefly, cells grown to 100% confluence in chambered culture slides were fixed in 4% paraformaldehyde for 10 minutes on ice and allowed to air-dry. Blocking was done in PBS containing 10% BSA for 30 minutes at 4°C. Incubation with primary antibody diluted in PBS containing 1% BSA and 0.2% Triton X-100 was done for 1 hour at room temperature. Incubation with secondary antibody (Alexa Fluor 488-conjugated goat anti-mouse (Cat No. 4412s, Cell Signaling, Danvers, MA) diluted 1:200 in PBS was done for 1 hour at room temperature. DNA was counterstained with Hoechst 33258 for 1 minute at room temperature. Preparations were mounted with Fluoromount-G. Images were acquired at 40X with an EVOS FL Imaging System for fluorescence and transmitted light applications (Cat. No. 6500-FL, Life Technologies, Waltham, MA) equipped with an integrated camera and EVOS Imaging System software.

### Immunoblotting

Immunoblotting was done following standard procedures [21]. Briefly, cells we lysed in 200 μL of RIPA buffer (Cat. No. 20–188, Sigma-Aldrich, St Louis, MO) with protease and phosphatase inhibitor cocktails (Cat. No. P8340-5ML, Sigma-Aldrich, St Louis, MO). Lysate protein concentration was determined by a Bradford protein quantification assay with the Bio-Rad Protein Assay Dye Reagent Concentrate (Cat. No. 500–0006, Bio-Rad, Hercules, CA). Thirty μg of protein were separated in a 10% polyacrylamide SDS-PAGE, transferred to a nitrocellulose membrane (Cat. No. 162–0115, Bio-Rad, Hercules, CA) for 1 hour and then immunoblotted overnight with primary antibody diluted in 10 ml of TBST containing 0.5 grams of BSA and 30 μl of sodium azide (the BSA was omitted when using phosphor-antibodies). Next day, membranes were incubated for 1 hour at room temperature in secondary antibody diluted in TBST. For signal development, we used the West Pico Chemiluminiscent Substrate Kit (Cat. No. 34078, ThermoFisher, Waltham, MA) exactly following manufacturer´s instructions. For image capture, we used Bio-Rad´s ChemiDoc Universal Hood II (Cat. No. 721br01236, Bio-Rad, Hercules, CA), using 120 seconds for image capture. Quantification of immunoblots band intensity was done using the ImageJ plug-in. Statistical non-parametric analyses were unpaired Mann-Whitney tests when two groups were compared and Kruskal-Wallis tests when three or more groups were compared.

### Immunohistochemistry and scoring

Immunohistochemistry was performed on purchased tissue microarrays following a previously published protocol [22]. Briefly, tissue slides were de-paraffinized in xylene and rehydrated in 100%, 95%, 80% and 75% ethanol washes, followed by a distilled water wash. Endogenous peroxidase was blocked with 3% hydrogen peroxide for 15 minutes at room temperature. Antigen retrieval was done for 40 minutes in citrate antigen retrieval solution that was preheated to 95–99°C, and then allowed to cool down for 30 minutes. Tissues were then incubated in primary antibody (or PBS for negative controls) overnight at 4–8°C in a sealed humid chamber. Tissues were then incubated with a biotin-conjugated secondary antibody solution for 30 minutes in the humid chamber. The secondary antibody was included in the Super Sensitive Link Label IHC kit (Cat. No. LP000-ULE, BioGenex, Fremont, CA); this same kit was used in subsequent steps for signal development following the manufacturer’s instructions. For slide mounting, tissues were dehydrated with 85%, 90%, 95% and 100% ethanol and a last incubation in xylene, oven-dried at 37°C for 30 minutes, and sealed with permanent mounting medium and coverslips. Slides that were used for pathology analyses were counterstained with hematoxylin-eosin. Immuno-stained slides were independently and blindly scored for the number of immune-positive cells by up to three pathologists, one of them scoring the whole tumor area to account for tumor heterogeneity and the others scoring at least four 40X visual fields per core. Pathologists used a 0 to 5 scoring system, in which a score of 0 meant 0% immune-positive cells per 40X visual field, and 1, 2, 3, 4 and 5 meant less that 1%, 1–10%, 11–33%, 34–66% and 67–100% immune-positive cells per 40X visual field, respectively. An additional scoring was conducted using the Aperio Image Software with the Positive Pixel Count algorithm. No data points or subjects were excluded from our analyses.

### Antibodies

Primary antibodies used for immunofluorescence, immunohistochemistry and immunoblotting were the following (if only one dilution is specified, this was for immunoblots): N-cadherin, 32/N mouse monoclonal, 1:1000 (Cat. No. 610920, BD Biosciences, San Jose, CA); Cdk5, 1:1000 (Cat. No. 2506s, Cell Signaling, Danvers, MA); E-cadherin, 24E10 mouse monoclonal, 1:000 for immunoblot and 1:800 for immunohistochemistry (Cat. No. 3195, Cell Signaling, Danvers, MA); Integrin α5, 1:1000 (Cat. No. 4705s, Cell Signaling, Danvers, MA); p35, C64B10 mouse monoclonal, 1:1000 (Cat No. 2680s, Cell Signaling, Danvers, MA); p39, 1:1000 for immunoblot (Cat. No. 3275s, Cell Signaling, Danvers, MA) and 1:50 for immunohistochemistry (Cat. No. ab124896, Abcam, Cambridge, MA); Rb, 4H1 mouse monoclonal, 1:1000 (Cat. No. 9309s, Cell Signaling, Danvers, MA); Vimentin, D21H3 mouse monoclonal, 1:1000 (Cat. No. 5741s, Cell Signaling, Danvers, MA); α/β tubulin, 1:1000 (Cat. No. 2148s, Cell Signaling, Danvers, MA); phospho-FAK (Y397), D20B1 Rabbit polyclonal, 1:1000 (Cat. No. 8556P, Cell Signaling, Danvers, MA); phospho-Rb (S249), rabbit polyclonal, 1:500 for immunoblot and 1:100 for immunohistochemistry (Cat. No. ab4788, Abcam, Cambridge, MA for immunoblots, and Cat. No. SAB 1305397–400 μL, Sigma-Aldrich, St. Louis, MO for immunohistochemistry); phospho-Rb (S807), rabbit polyclonal, 1:500 (Cat. No. ab192190, Abcam, Cambridge, MA); phospho-Rb (S807/S811) D20B12, rabbit polyclonal, 1:1000 (Cat. No. 8516s, Cell Signaling, Danvers, MA); phospho-Rb (T821), rabbit polyclonal E231, 1:500 for immunoblot and 1:100 for immunohistochemistry (Cat. No. ab32015, Abcam, Danvers, MA); phospho-Rb (S612), rabbit polyclonal, 1:500 (Cat. No. GTX24777, Genetex, Irvine, CA); phospho-Rb (S608), rabbit monoclonal D10F2, 1:1000 (Cat. No. 8147, Cell Signaling, Danvers, MA); phospho-Rb (S780), rabbit monoclonal D59B7, 1: 1000 (Cat. No. 8180, Cell Signaling, Danvers, MA); phospho-Rb (S795), rabbit polyclonal, 1: 1000 (Cat. No. 9301, Cell Signaling, Danvers, MA). Secondary antibodies for immunohistochemistry were part of the Super Sensitive Link Label IHC kit (Cat. No. LP000-ULE, BioGenex, Fremont, CA). Secondary antibodies for immunoblots were either goat anti-rabbit (Cat. No. 7074, Cell Signaling, Danvers, MA) or horse anti-mouse (Cat. No. 7076, Cell Signaling, Danvers, MA) and were diluted at 1:4000 in TBST.

### Quantitative reverse transcription PCR (qRT-PCR)

Total RNA was harvested from confluent cultures using the RNeasy Mini Kit (Cat. No. 74104, Qiagen, Redwood City, CA) following manufacturer´s instructions. RNAs were then reverse-transcribed using the iScript cDNA Synthesis kit (Cat. No. 1708890, Bio-Rad, Hercules, CA), also following the manufacturer´s instructions. Real time PCR was performed using Bio-Rad´s iQ SYBR Green Supermix (Cat. No. 1725120, Bio-Rad, Hercules, CA) on an Eppendorf Realplex 2 Real Time PCR apparatus. Briefly, 50 ng of cDNA were subjected to 50 amplification cycles, following the instructions provided by the different gene-specific Applied Biosystems (Foster City, CA) Taqman assays as follows: N-cadherin (Mm00483213_m1), E-cadherin (Hs00170423_m1); and Vimentin (Hs00958111_m1). Target gene expression was normalized to the GAPDH reference gene (Mm00483213, Applied Biosystems, Foster City, CA) and relative expression levels were determined using the ^ΔΔ^Ct method. Real time qRT-PCR data analysis was conducted by unpaired t-test evaluations of cycle threshold (Ct) values, defined as the number of PCR cycles required for the fluorescent signal to exceed the background signal level.

### Tridimensional hanging drop spheroid protocol

This cell-to-cell adhesion assay was performed as previously described without any modifications [23–25]. Briefly, H1666 and H520 cells were serum-starved for 6 hours and detached from confluent cultures with 0.25% trypsin- EDTA solution for 10 minutes at 37°C. The single cell suspensions were transferred to 15 mL conical tubes. The hanging drop suspension set-up was assembled using 100 mm culture dishes in which 5 mL of sterile PBS was added to the bottom of the dish to generate an interior humid atmosphere. Twenty-seven μL drops of cell suspension were placed at various points in the inner surface of the lid, placing 20 of such drops per lid, with sufficient space between drops to avoid their merging. The lids were then placed on the PBS-containing plate in such a manner that the cell suspension drops remained hanging from the lid and plates were incubated at 37°C and 5% CO_2_, until they were collected for photomicrography at 2, 4, 24 and 48 hours. Photomicrographs of the hanging drops were taken at 10X using an EVOS FL fluorescence/transmitted light Imaging System (Life Technologies, Waltham, MA). Data quantification and analyses were performed from 10 hanging droplets. Identification of spheroids was done using NIH´s ImageJ software on 32-bit images taken with the EVOS FL microscope. Spheroid size and discrimination between shapes was achieved using the ImageJ Particle Analysis Plug-in and was done based on circularity of the shape (from 0 to 1) and spheroid size as calculated by the plug-in based on pixel density (from 200 to 3,000), and determination of the total area for each spheroid. Quantification of the number of spheroids per 10X visual field was done using the ImageJ Cell Counter Analysis Plug-in. Statistical analyses were Kruskal-Wallis and differences with p-values of less than 0.05 were considered statistically significant.

### Mass spectrometry

Total Rb protein was purified from H1666 and H520 cells by running protein lysates in an SDS-PAGE followed by gel excision of bands of 105 kDa molecular weight. Disulfide bonds were reduced with tris-carboxy-ethyl-phosphine and free cysteines were alkylated with iodoacetamide prior to in-gel digestion with trypsin. Three Rb purifications were prepared from biological replicates for each cell line and analyzed with LC-MS/MS (U3000, Dionex and LTQ-Orbitrap, Thermo). Database searches were conducted against human entries in the UniProt database using Mascot (Matrix Science) and Sequest (Thermo); results were combined in Scaffold (Proteome Software). Ion chromatograms for phosphopeptide quantification were extracted using QualBrowser (Thermo).

### Bovine intestinal alkaline phosphatase (BIP) treatment

Protein lysates were prepared and quantified as described above. For treating lysates with BIP (Cat. No. P0114, Sigma-Aldrich, St. Louis, MO), we follow the protocol accompanying the product. Briefly, 400 μg of total protein were treated with 123.8 U of BIP in dephosphorylation buffer (0.5 mmol/L Tris-HCl, pH 7.9, 10 mmol/L NaCl, 1 mmol/L MgCl2, 0.1 mmol/L DTT) for 30 minutes at 30°C. For negative controls we used the BIP inhibitors 50 mM sodium orthovanadate (Cat. No. S6508-10G, Sigma-Aldrich, St Louis, MO), 5 mM sodium pyrophosphate (Cat. No. 221368-100G, Sigma-Aldrich, St Louis, MO), and 100 mM sodium fluoride (Cat. No. S1504-100G, Sigma-Aldrich, St Louis, MO). Immunoblotting of BIP-treated protein lysates using the anti-phospho-Rb S249 antibody was done exactly as described above. Statistical non-parametric analyses of quantified band intensities were unpaired Mann-Whitney tests when two groups were compared and Kruskal-Wallis tests when three or more groups were compared.

### Adenovirus-driven p39 expression

H1666 cells cultures in RPMI medium were plated in 6-well plates until a confluence of 70–80%. Cells were infected with either adenoviral vectors carrying the p39 gene (Ad-GFP-h-CDK5R2, Cat. No. ADV-204851, Vector Biolabs, Malvern, PA) or a scrambled sequence control adenoviral vector (Ad-U6-RNAi-GFP, Cat. No. 1122N, Vector Biolabs, Malvern, PA). Infections were done at a multiplicity of infection (MOI) of 1,000 viral particles per cell. Cells were harvested 24 hours after infection and evaluated for p39 expression by immunoblot analysis.

### Tumor microarrays

Lung cancer tumor microarrays (TMAs) with formalin fixed, paraffin embedded tissues mounted on positively charged SuperFrost Plus glass slides were obtained from US Biomax, with catalog numbers LC241c, LC488, and T041 (US Biomax, Derwood, MD). The TMA LC241c contains 24 biospecimens cores including 12 lung adjacent normal tissues, 2 adenocarcinomas, and 10 squamous cell carcinomas. The TMA LC241b contains biospecimens from 24 patients containing 12 lung adjacent normal tissues, 2 adenocarcinoma, and 10 squamous cell carcinomas. The TMA T041 contains biospecimens from 24 patients containing 8 normal lung tissues, 4 adenocarcinoma, 4 large cell carcinoma, 4 small cell carcinoma, and 4 squamous cell carcinomas. The TMA LC488 contains biospecimens from 48 patients containing 12 adenocarcinoma, 10 lung squamous cell carcinoma, 4 papillary adenocarcinoma, 2 large cell carcinoma, 4 small cell carcinoma, and 16 cancer adjacent tissue. The tissue microarray tissue cores were 5 microns in thickness, 0.6–1.0 mm in diameter and spaced at 0.25 mm. US Biomax supplied the following clinicopathologic data for each core: patient gender and age, tumor histological grade, TNM staging, lymph node metastases, and metastases to distant organs.

### Statistical analyses of immunohistochemistry scoring

Spearman correlation analyses done with the GraphPad Prism 7 software were conducted to assess correlations between IHC staining for phosphorylated Rb, E-cadherin, and p39 and any of the clinical parameters accompanying the tissue cores. Lung cancer tissue microarrays were scanned and uploaded into Aperio Scope (Aperio Technologies, Inc., Vista, CA) and analyzed using the Aperio ImageScope Positive Pixel Count (PPC) algorithm. The positivity (number of positive pixels over total number of pixels) of each sample was obtained for statistical analysis for phospho-Rb S249 and phospho-Rb T821, E-cadherin, and for p39 expression. A linear regression model with Minitab Statistical Software version 17 was conducted to assess the possible association between lung squamous cell lung cancer staging and the positivity scores for phospho-Rb S249 and phospho-Rb T821, E-cadherin, and for p39 expression. Variables with significant association on ANOVA analysis were entered into a linear regression model; the p-value threshold to enter/leave the model was set to 0.10. The Levene test was used to assess the equality of residual variances. The Runs test was used to evaluate the randomness of the residuals and the Kolmogorov-Smirnov test was implemented to verify if the residuals are normally distributed. A simple and easy clinical lung cancer response prediction formula, which produces a score for each subject ranging from 1 to 4, was developed using the estimated coefficients of the variables in the final model. A power analysis to estimate the necessary number of patients for experiments was calculated based on data from all the IHC staining of TMAs documented in this report, consisting of a total of 58 lung cancer patients from three lung tissue microarrays. It was conducted using Minitab´s Power and Sample Size. The power analyses yielded that the minimum sample size needed to obtain a power that of at least 80% is 60 patients, and for a power of 75% is 50 patients. Performance of the IHC scores on a continuous scale was analyzed by Receiver Operator Characteristics (ROC) curves, in which the area under the curve ranges between 0.5, indicating no predictive value, to 1.0, indicating perfect predictive accuracy. For each scoring, sensitivity and specificity were calculated against the gold standard represented by the morphologic diagnosis. Sensitivity was defined as the proportion of subjects that have the target condition and also give positive test results [26], while specificity was defined as the proportion of subjects without the target condition and give negative results [26]. The ROC curves were generated using GraphPad Prism 7, and graphically displays the trade-off between sensitivity and specificity.

## Results

### H520 cells shows traits of epithelial-to-mesenchymal transition

**Fig 1:**
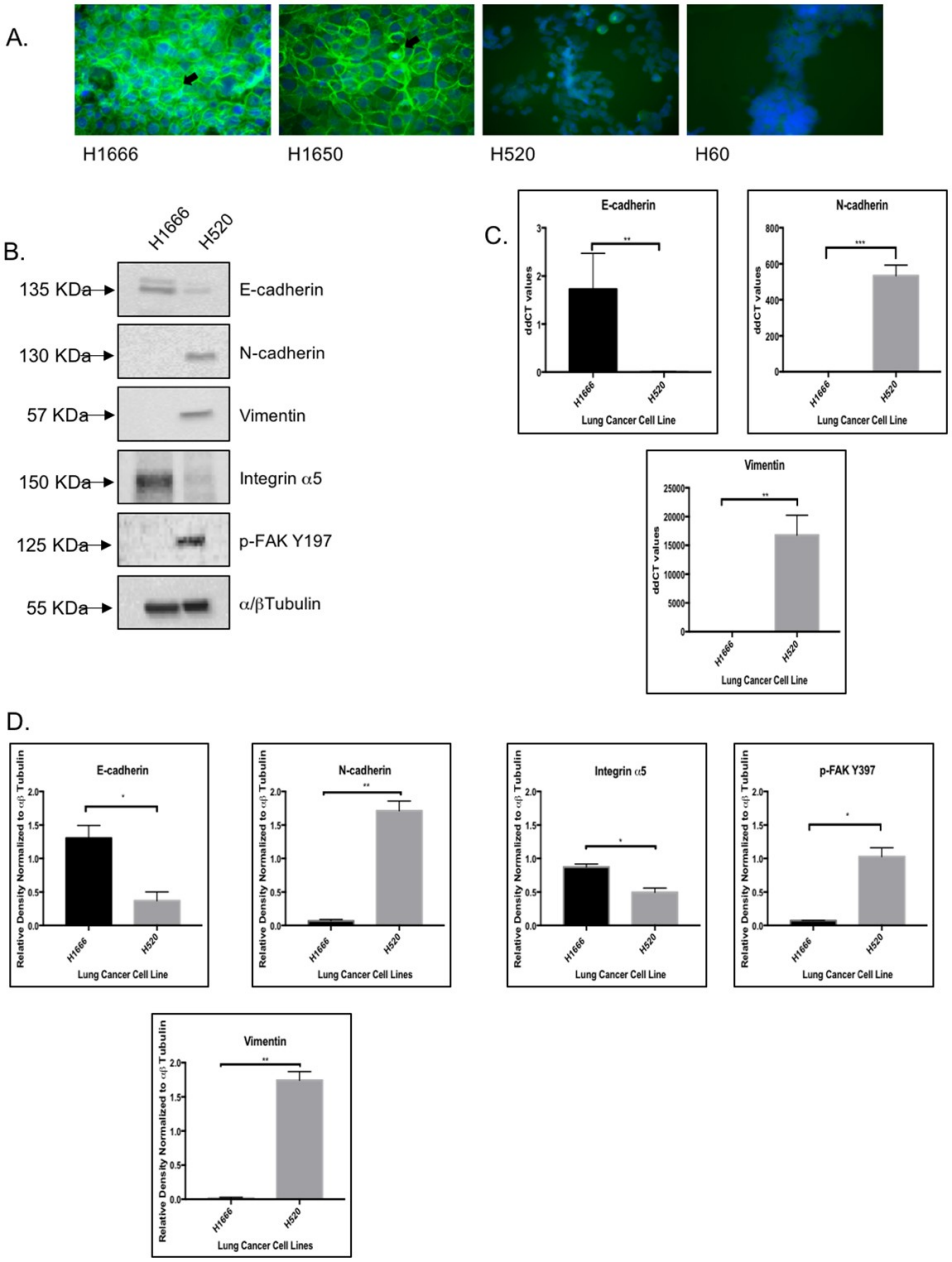
The H520 cell line lacks adherens junctions and expresses EMT traits. (A) E-cadherin immunofluorescence showing membrane-associated E-cadherin localization (*arrows*) in H1666 and H1650 cells. In contrast, E-cadherin expression is notably decreased in H520 cells in a diffuse cytoplasmic staining pattern. E-cadherin expression is undetectable in SCLC H60 cells. In all preparations nuclei were counterstained with DAPI (blue). (B) Representative immunoblots showing decreased E-cadherin, increased N-cadherin and vimentin, decreased integrin α5 expression and increased FAK phosphorylation on Tyr397 in H520 cells relative to H1666 cells. Immunoblot using an anti α/β tubulin antibody (*bottom panel*) shows equal loading of the gel. (C) EMT marker expression was also measured at the mRNA level by qRT-PCR. While levels of the E-cadherin transcript are elevated in H1666 relative to H520 cells, the levels of N-cadherin and vimentin transcripts are elevated in H520 cells relative to H1666 cells, in all three instances in a statistically significant manner. Error bars are standard error of the mean, and P-values are shown on top of each graph. (D) Immunoblot replicas were quantified by densitometric analyses of band intensity. Bars represent band intensity for each protein, normalized against the α/βtubulin loading control, and error bars are standard error of the mean. P values, shown on top of each graph, indicate that all the observed changes in protein expression were statistically significant. Statistical tests were assessed by Mann-Whitney tests when comparing two groups and Kruskal Wallis when comparing three groups or more. Statistical significance was defined as, *p-value < 0.05, **p-value < 0.01, and ***p-value < 0.001.

We conducted an immunofluorescence (IF) analysis to assess the expression and localization of E-cadherin in a panel of NSCLC cell lines consisting of the H1666 and H1650 AC cell lines and the H520 SCC cell line. The panel also included the small cell lung carcinoma (SCLC) cell line H60, known for being Rb-null. As can be seen in Fig 1A, H1666 and H1650 cells show a strong and membrane-associated E-cadherin staining, labeling being more intense in H1666 cells. E-cadherin is seen along borders of cell-to-cell contact, suggestive of adherens junction structures. In contrast, H520 cells show weak and diffuse cytoplasmic E-cadherin staining comparable to the Rb-deficient SCLC H60 cell line.

We focused our subsequent studies on H1666 and H520 cells, in which we studied the expression of EMT markers by immunoblot and qRT-PCR analyses. As shown in Fig 1B and 1C, H520 cells show diminished E-cadherin expression relative to H1666 cells both at the protein (Fig 1B) and mRNA levels (Fig 1C), consistent with the decreased, diffuse E-cadherin localization in H520 cells (Fig 1A). Conversely, N-cadherin levels are increased in H520 cells relative to H1666 cells (Fig 1B and 1C), indicative of the cadherin switch known to occur during EMT. Increased vimentin expression, another EMT trait, is also observed in H520 cells relative to H1666 (Fig 1B and 1C). We also checked for markers of cell-to-substrate adhesion by immunoblot analysis. Expression of integrin α5, a subunit of the fibronectin receptor, is diminished in H520 cells relative to H1666 cells, while the opposite is observed for phosphorylated focal adhesion kinase (FAK), a cytosolic protein tyrosine kinase that connects integrins with the actin cortical skeleton, and whose activation triggers cell motility and invasion [27] (Fig 1B). In the FAK western blot, we used an antibody that detects FAK phosphorylation on Tyr 397, which is required for FAK activation and increases in association with loss of cell-to-substrate adhesion, increased motility and invasive capacity, as well as correlating with tumor staging in several cancer types [27]. All immunoblot results were subjected to quantification by densitometric analyses and normalized against α/β beta tubulin (Fig 1D). Taken together, our data show the manifestation of EMT markers in H520 cells relative to H1666 cells.

### H520 cells have impaired cell-to-cell adhesion

**Fig 2:**
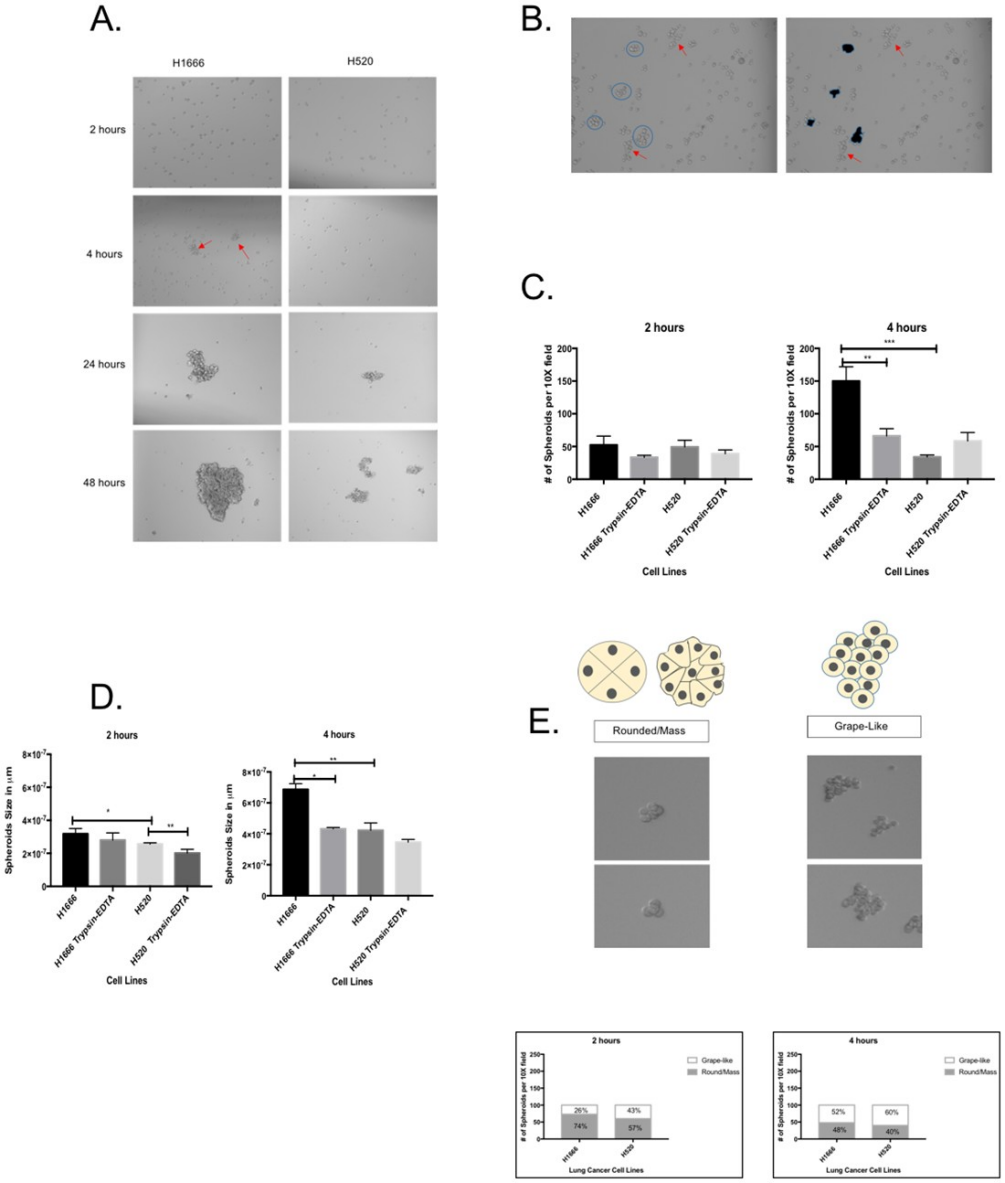
H520 cells have impaired cell-to-cell adhesion. (A) Photomicrographs of hanging drop H1666 and H520 cultures taken at 10X at 2, 4, 24, and 48 hours. Spheroids first appear at 4 hours in H1666 cells (*red arrows*), while in H520 cells they form at 24 hours, a time point at which H1666 cells already exhibit enlarged spheroids. (B) Photomicrograph of a H1666 cells hanging drop culture taken at 10X illustrating the spheroids identified by the ImageJ Particle Analysis Plug-in. Some aggregates are not detected as spheroids by ImageJ due to low compaction factor (*red arrows*), which denotes a lack of strong adhesion. (C) Graphs showing the total number of spheroids per 10X visual field at 2 and 4 hours. Control cultures were grown on 0.4% trypsin-EDTA to inactivate cadherin-based adhesion. (D) Graphs showing spheroid size in H1666 and H520 cells. No large difference between H1666 and H520 cells was detected at 2 hours, while at 4 hours H1666 spheroids were significantly larger than in H520 cells, with the size increase being reduced by 0.4% trypsin-EDTA. (E) At 2 hours the adhesive rounded/mass spheroid morphology predominates in H1666 cells, while the less adhesive grape-like morphology predominates in H520 cells. This trend continues at 4 hours. Statistical tests were assessed by Mann-Whitney tests when comparing two groups and Kruskal Wallis when comparing three groups or more. Statistical significance was set as: *p-value < 0.05, **p-value < 0.01, and ***p-value < 0.001. The cartoons were modified from Kenny et al., 2007.

Given that H520 cells have decreased E-cadherin expression, lack adherens junction structures and express EMT markers, we wanted to investigate if these cells have diminished cell-to-cell adhesion. For this purpose, we used a 3D hanging drop spheroid formation assay exactly as previously described [23–25]. This assay measures the capacity of cells suspended in medium to form spheroids, which is dependent on cell adhesion. After serum-starvation for 6 hours to rule out the effect of cell proliferation in spheroid formation, we monitored spheroid formation under a microscope at 2, 4, 24 and 48 hours after experiment initiation. Fig 2A shows representative 10X phase contrast photomicrographs of cultures. As can be observed, cultures are still single-cell suspensions in both H1666 and H520 cell lines 2 hours after initiating the assay, but H1666 cells start to form spheroids as early as at 4 hours (*red arrows*), while it takes 24 hours for the first small spheroids to appear in H520 cell cultures. Spheroids continue to grow in size in H1666 cells at 24 and 48 hours, while spheroid size in H520 cells remain comparatively small even at 48 hours. We repeated the spheroid assays in the presence of trypsin-EDTA to confirm that adhesion is cadherin-mediated, as this type of adhesion is known to be calcium dependent [28]. For quantification of the number of spheroids per 10X visual field and measuring their size in μm we used the ImageJ software in conjunction with a cell counter analysis ImageJ Plug-In as previously described [29]. Fig 2B shows a 10X photomicrograph of H1666 cells illustrating the spheroids as identified by ImageJ. Some cellular aggregates are not detected as spheroids by ImageJ (indicated by red arrows) in spite of being clusters. Even though they are comprised of several cells, rather than forming spheroids, they form sheet-like structures lacking a strong compaction factor, which in this assay denotes a lack of strong adhesive properties, and therefore we did not program ImageJ to detect sheet-like cellular aggregates. We only quantified the data at 2 and 4 hours after setting-up the hanging drop cultures since at this time points cell proliferation is unlikely to be a significant influence for spheroid formation. The graphs in Fig 2C show the total number of spheroids formed by H1666 and H520 cells at 2 and 4 hours, with and without Trypsin-EDTA. Again, no appreciable differences in the number of spheroids/10X field can be observed between H1666 and H520 cells after 2 hours in culture. Statistically significant differences between H1666 and H520 cells become apparent at 4 hours in culture, when H1666 cells form more than thrice as many spheroids/10X field than H520 cells, an increase that is abrogated by trypsin-EDTA. When evaluating the spheroid size in H1666 and H520 cells, no differences in size between H1666 and H520 cells were apparent at 2 hours, differences becoming noticeable at 4 hours, when H1666 cell spheroids were significantly larger than those formed by H520 cells, with spheroid size being reduced by trypsin-EDTA treatment (Fig 2D).

Finally, we also studied spheroid morphology which is highly informative of the adhesive properties of its constituent cells. ImageJ enabled us to distinguish between two basic spheroid morphologies, namely, rounded/mass and grape-like structures. These morphologies are illustrated in the cartoons at the top of Fig 2E and the accompanying photomicrographs. Cells in spheroids with a rounded/mass morphology are considered to be more adhesive, while cells in grape- or sheet-like spheroids tend to have diminished adhesive properties [23–25]. As shown in Fig 2E, at two hours, even though H1666 and H520 do not differ dramatically in the number of spheroids per 10x visual field, H1666 spheroids are predominantly rounded/mass (74%), while in the H520 cells the spheroids are predominantly grape-like (57%). At 4 hours H1666 cells show roughly equal proportions of rounded/mass versus grape-like spheroids, while H520 cells show a predominance of the poorly adhesive grape-like spheroid morphology (60%). In summary, our data show that, relative to H1666 cells, H520 cells lack epithelial cell adhesion markers, express EMT markers, and have impaired cell-to-cell adhesion capacity.

### H520 cells show increased Rb phosphorylation on Serine 249 and Threonine 821 relative to H1666 cells

**Fig 3:**
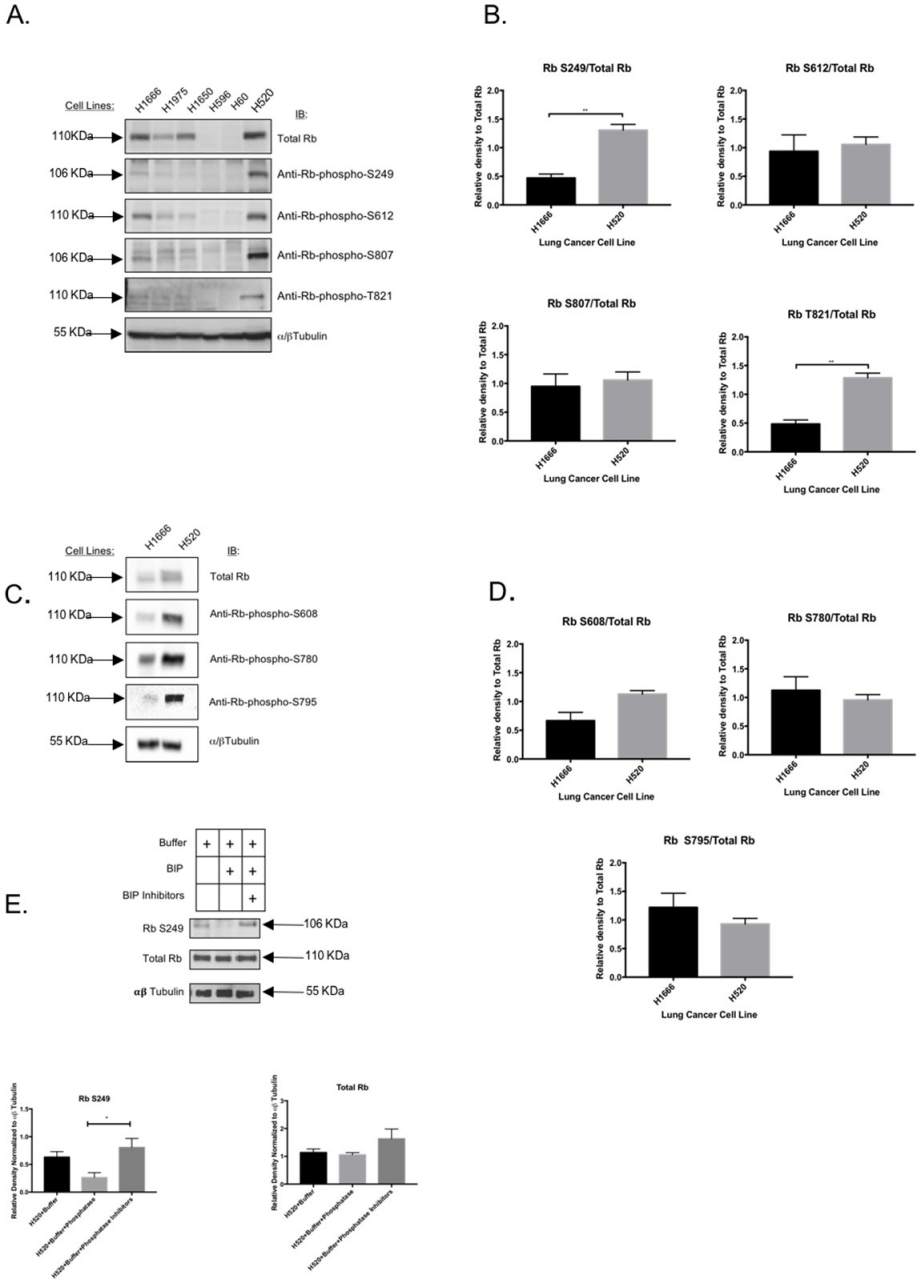
H520 cells show increased Rb phosphorylation on S249 and T821 relative to H1666 cells. (A) Immunoblot validation of LC-MS/MS data showing phosphorylation of Rb residues S249, S612, S807, and T821 in a panel of lung cancer cell lines. These residues were shown by LC-MS/MS to be hyper-phosphorylated in H520 cells relative to H1666 cells. The bottom lane shows a blot for alpha-beta tubulin as a loading control. (B) These immunoblots were conducted in triplicates and quantified by densitometric analyses of band intensity using the ImageJ Plug-in. Each data point normalizes the Rb phosphorylation signal against total Rb expression. Error bars are standard error of the mean. Only the Rb phosphorylation on S249 and T821 were increased in H520 cells relative to H1666 cells in a statistically significant manner. (C) and (D) Immunoblot analyses (C) and quantification of band intensity (D) of Rb phosphorylated residues which were not detected by mass spectroscopy to be differentially phosphorylated between H1666 and H520 cell lines. Each data point represents the band intensity of the phosphorylated Rb residue normalized against total Rb expression. None of these residues were differentially phosphorylated between H1666 and H520 cells in a statistically significant manner. (E) Whole protein extracts from H520 cells were treated with bovine intestinal alkaline phosphatase (BIP) buffer alone (first immunoblot lane), with BIP in phosphatase buffer (middle immunoblot lane) and with BIP in its buffer plus BIP inhibitors (third immunoblot lane). BIP treatment diminished the intensity of the Rb S249 band in a statistically significant manner without affecting total Rb levels. This effect was reverted in the presence of BIP inhibitors. In all quantification data, error bars are standard error of the mean, and statistical tests were Mann-Whitney tests when comparing two groups and Kruskal Wallis when comparing three groups or more. Statistical significance was set as: *p-value < 0.05, **p-value < 0.01, and ***p-value < 0.001.

In most human cancers Rb is inactivated by a chronic hyper-phosphorylation that abolishes its role as a cell cycle regulator [2,3,7,17–19,30,31]. However, no evidence currently links Rb hyper-phosphorylation to defective cell adhesion and EMT traits. Given the presence of EMT traits in H520 cells, we hypothesized that H520 cells possess a “metastasis- or EMT-associated Rb phosphorylation signature”. To test this, we conducted a liquid chromatography-tandem mass spectroscopy (LC-MS/MS) analysis of Rb purified from both H1666 and H520 cells to detect global differences in Rb phosphorylation between them. We started by immunoprecipitating total Rb from both H1666 and H520 cell lines, followed by SDS-PAGE of the immunoprecipitated Rb. The resolved Rb band from each cell line was gel-purified and subjected to LC-MS/MS peptide sequencing, phosphorylation site mapping, and relative quantification, which was conducted from three separate Rb purifications from each cell line. Our triplicate analysis showed that the Rb residues S249, S612, S807, and T821 were hyper-phosphorylated in H520 cells relative to H1666 cells. We validated the LC-MS/MS data by immunoblotting using phospho-specific antibodies. Fig 3A shows a representative immunoblot (from triplicates) conducted in a panel of lung cancer cell lines, performed using antibodies that recognize total Rb, anti-Rb-phospho-S249, anti-Rb-phospho-S612, anti-Rb-phospho-S807, and anti-Rb-phospho-T821, using α/β tubulin as a loading control. As can be seen, H520 cells (*sixth lane*) show slightly elevated levels of total Rb relative to H1666 cells (*first lane*), as well as increased phosphorylation in Rb residues S249, S612, S807 and T821. We quantified band intensities by densitometric analysis followed by statistical analyses using GraphPad PRISM and normalized the band intensity of each anti-Rb-phospho blot against the band intensity for total Rb levels. The graphs in Fig 3B show that only residues S249 and T821 are hyper-phosphorylated in H520 cells relative to H1666 cells in a statistically significant manner, with p-values of 0.004 and 0.002, respectively. We also immunoblotted for the phosphorylation of other Rb residues not shown by our LC-MS/MS analysis to be differentially phosphorylated between H1666 and H520 cells, such as S608, S780, and S795. As can be seen in Fig 3C and 3D, none of these residues significantly differed in phosphorylation state between H1666 and H520 cells after quantification and statistical analyses. Finally, to ensure that the immunoreactivity generated by our antibody is specific for a phosphorylated version of S249, we treated protein lysates from H520 cells with a phosphatase enzyme. As shown in Fig 3E, phosphatase treatment abolished the immunoreactivity generated by the phosho-Rb S249 antibody without affecting total Rb levels. In summary, H520 cells, which show decreased adhesive properties and EMT traits, appear to have increased phosphorylation on Rb residues S249 and T821, relative to H1666 cells.

### H520 cells have increased levels of the Cdk5 activator p39

**Fig 4:**
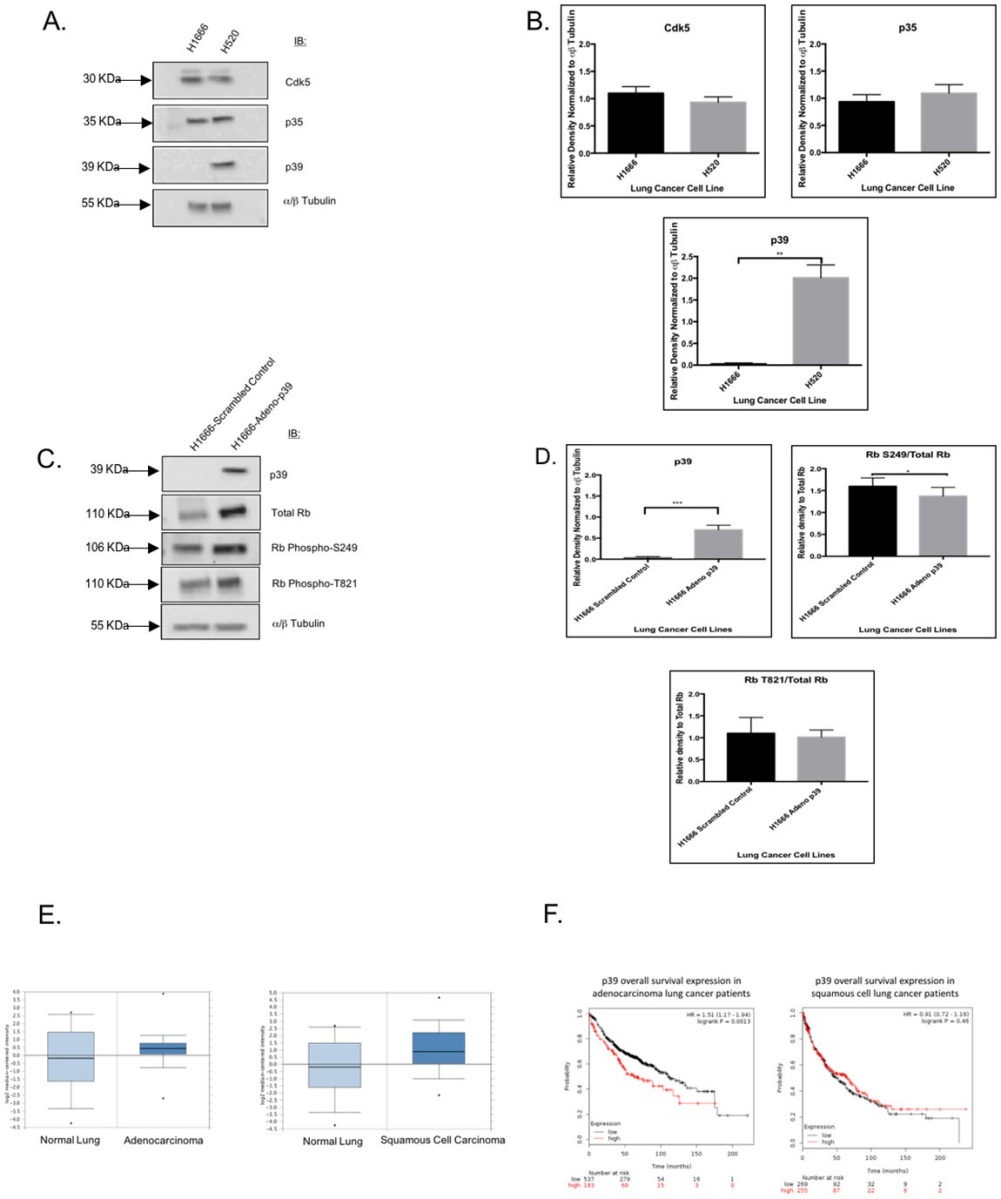
H520 cells have increased levels of the Cdk5 activator p39. (A) Immunoblots showing comparable levels of Cdk5 and p35 in H1666 and H520 cells. However, H520 cells have increased expression of the second major Cdk5 activator p39, relative to H1666 cells. Alpha-beta tubulin was blotted as a loading control. (B) After quantification of band intensity in triplicate immunoblots only p39 expression resulted to be increased in a statistically significant manner in H520 cells relative to H1666 cells (p-value of 0.007) based on an unpaired t-test statistical analysis. (C) Immunoblots showing that phosphorylation levels of Rb S249 and T821 were not significantly affected upon reintroduction of p39 into H1666 cells, in spite of a robust p39 expression in H1666 cells 24 hours after adeno-p39 infection relative to cells transfected with an adenovirus bearing a scrambled p39 sequence. (D) Band intensities in the triplicate immunoblots shown in **C**. were quantified using the ImageJ Plug-in and phosphorylation in Rb S249 and T821 was graphed after normalization against total Rb. No significant effect on Rb phosphorylation on S249 or T821 upon p39 forced expression in H1666 cells can be detected, with p-values shown at the top of each graph. (E) NSCLC microarray expression data from the Oncomine database (http://www.oncomine.org, Compendia Bioscience, Ann Arbor, MI, USA), specifically from the study conducted by Bhatacharjee et al. This study contained 139 lung adenocarcinomas, 21 squamous cell carcinomas, 6 small cell lung carcinomas, and 17 normal lung tissues. Datasets were ordered by overexpression of CDK5R2 (p39), which was analyzed by Affymetrix U95A microarrays (Affymetrix ID 36361_at). Box plots show that p39 is over-expressed 1.76-fold in AC relative to normal lung (p-value of 0.060) and that the p39 overexpression is more pronounced in SCC by 2.71-fold relative to normal lung (p-value of 0.012). (F) Kaplan Meier plots showing reduced overall survival of AC patients with higher expression of p39 (p-value of 0.0013), the effect being less pronounced and non-statistically significant in SCC patients. The plots were generated using the KM Plotter Online Tool (http://www.kmplot.com), which relates microarray expression data (Affymetrix ID: 20582_at, in this case for p39 expression) with clinical outcomes. Statistical tests were assessed by Mann-Whitney tests when comparing two groups and Kruskal Wallis when comparing three groups or more. Statistical significance was set as: *p-value < 0.05, **p-value < 0.01, and ***p-value < 0.001.

We next attempted to identify a kinase that could phosphorylate Rb on S249 and T821, focusing on cyclin-dependent kinase 5 (Cdk5) as a potential candidate. We started with immunoblots to study the expression of Cdk5 and its activators CdkR1 (p35) and CdkR2 (p39) in H1666 and H520 cells. Contrary to our expectations, H1666 and H520 cells did not differ in Cdk5 and p35 levels (Fig 4A and 4B). However, we did observe a dramatic increase in p39 in H520 cells relative to H1666 cells (Fig 4A and 4B). To determine if p39 is involved in the generation of the Rb S249/T821 phosphorylation signature, we forced p39 expression in H1666 cells by viral infection. Fig 4C and 4D show that in spite of robustly induced p39 expression in p39-infected H1666 cells, Rb phosphorylation on S249 and T821 did not significantly increase relative to scrambled control-infected H1666 cells. Although Cdk5/p39 may not be the kinase complex that engenders the Rb S249/T821 phosphorylation signature, we still maintained an interest in p39 as a potential metastasis marker. According to the Bhattacharjee lung cancer dataset in *ONCOMINE*, p39 mRNA expression is elevated in lung AC and SCC relative to normal lung, with p-values of 0.060 and 0.012, respectively (Fig 4E). Kaplan Meier plots show that lung AC patients with high tumor gene expression of p39 have a significantly reduced overall survival (p-value = 0.0013) relative to patients with low tumor p39 expression (Fig 4F). Further evaluation of clinical data revealed that lung cancer patients whose tumors have an EMT-associated gene expression profile have higher p39 levels than patients whose tumors lack such a signature (S1A and S1B Fig and S1 Table).

### Increased phosphorylation on Rb S249 correlates with advanced grading while increased p39 staining correlates with increased tumor staging, lymph node invasion, and distant metastasis

We next assessed the clinical value of Rb S249 and T821 phosphorylation and of p39 expression by immunohistochemical (IHC) staining of lung cancer tumor microarrays (TMAs) followed by establishing correlations with the clinical data accompanying each tumor core. We obtained three commercial lung cancer TMAs (TMA1 LC241C, TMA2 LC488, and TMA3 T041 from US Biomax, Inc.), and independently IHC-stained them with antibodies against phospho-Rb-S249, phospho-Rb-T821 and p39. In addition to lung tumor tissue, each TMA included controls of either tumor-adjacent normal lung tissues or of benign lung diseases. Each core in the TMAs had accompanying clinical data, such as tumor stage, grade, size, and spread to lymph nodes or distant metastases, which we correlated with Rb S249 and T821 phosphorylation and with p39 expression. A detailed description of each TMA can be found in Tables 1, 2 and 3.

**Table 1:** Complete description of TMA LC241C.

Core TMA1 LC241C	Histological Type	Age	Sex	Grade	Stage	Size	Lymph Node Metastases	Distant Metastases
A1	Squamous Cell Carcinoma	64	M	3	4	3	1	1
A2	Squamous Cell Carcinoma	64	M	3	4	3	1	1
A3	Adjacent Normal	64	M	-	-	-	-	-
A4	Adjacent Normal	64	M	-	-	-	-	-
A5	Squamous Cell Carcinoma	66	M	2	1	2	0	0
A6	Squamous Cell Carcinoma	66	M	2	1	2	0	0
A7	Pulmonary edema with congestion	66	M	-	-	-	-	-
A8	Pulmonary edema with congestion	66	M	-	-	-	-	-
B1	Squamous Cell Carcinoma	67	M	3	2	3	0	0
B2	Squamous Cell Carcinoma	67	M	3	2	3	0	0
B3	Adjacent Normal	67	M	-	-	-	-	-
B4	Adjacent Normal	67	M	-	-	-	-	-
B5	Squamous Cell Carcinoma	49	M	3	2	2	1	0
B6	Squamous Cell Carcinoma	49	M	3	2	2	1	0
B7	Adjacent Normal	49	M	-	-	-	-	-
B8	Adjacent Normal	49	M	-	-	-	-	-
C1	Squamous Cell Carcinoma	53	M	3	1	2	1	0
C2	Squamous Cell Carcinoma	53	M	3	1	2	0	0
C3	Adjacent Normal	53	M	-	-	-	-	-
C4	Adjacent Normal	53	M	-	-	-	-	-
C5	Adenocarcinoma	42	M	3	1	2	0	0
C6	Adenocarcinoma	42	M	3	1	2	0	0
C7	Adjacent Normal	42	M	-	-	-	-	-
C8	Adjacent Normal	42	M	-	-	-	-	-
-	Adrenal Gland Pheochromocytoma (tissue marker)	42	M	-	-	-	-	-

Each core indicates the tissue type, patient´s age and sex, and tumor grade, stage, size, and metastases to lymph nodes and to distant sites.

**Table 2:** Complete description of TMA LC488.

**Core TMA2 LC488**	**Histological Type**	**Age**	**Sex**	**Grade**	**Stage**	**Size**	**Lymph Node Metastases**	**Distant Metastases**
A1	Adenocarcinoma	64	F	2	1	1	0	0
A2	Adenocarcinoma	51	M	2	1	2	0	0
A3	Adenocarcinoma	37	F	2	1	2	0	0
A4	Adenocarcinoma	67	M	3	1	2	0	0
A5	Adenocarcinoma	46	M	2	1	2	0	0
A6	Adenocarcinoma	63	F	2	1	2	0	0
A7	Adenocarcinoma	57	M	2	1	2	0	0
A8	Adenocarcinoma	56	M	3	1	2	0	0
B1	Adenocarcinoma	64	F	2	1	2	0	0
B2	Adenocarcinoma	51	M	2	1	2	0	0
B3	Adenocarcinoma	37	F	2	1	2	0	0
B4	Adenocarcinoma	67	M	3	1	2	0	0
B5	Adenocarcinoma	46	M	2	1	2	0	0
B6	Adenocarcinoma	63	F	2	1	2	0	0
B7	Adenocarcinoma	57	M	2	1	2	0	0
B8	Adenocarcinoma	56	M	3	1	2	0	0
C1	Adjacent Normal	64	F	-	-	-	-	-
C2	Adjacent Normal	51	M	-	-	-	-	-
C3	Adjacent Normal	37	F	-	-	-	-	-
C4	Adjacent Normal	67	M	-	-	-	-	-
C5	Adjacent Normal	46	M	-	-	-	-	-
C6	Adjacent Normal	63	F	-	-	-	-	-
C7	Stromal hyperplasia with congestion and edema	57	M	-	-	-	-	-
C8	Adjacent Normal	56	M	-	-	-	-	-
D1	Squamous cell carcinoma	60	M	2	1	2	0	0
D2	Squamous cell carcinoma	44	M	1	2	2	1	0
D3	Squamous cell carcinoma	54	M	2	3	2	3	0
D4	Squamous cell carcinoma	60	M	3	2	2	3	0
Core TMA2 LC488	Histological Type	Age	Sex	Grade	Stage	Size	Lymph Node Metastases	Distant Metastases
D5	Squamous cell carcinoma	64	M	2	1	2	0	0
D6	Large cell carcinoma	55	M	0	1	2	0	0
D7	Small cell carcinoma	56	M	0	1	2	0	0
D8	Small cell carcinoma	43	M	0	1	2	0	0
E1	Squamous cell carcinoma	60	M	2	1	2	0	0
E2	Squamous cell carcinoma	44	M	1	2	2	1	0
E3	Squamous cell carcinoma	54	M	2	3	2	3	0
E4	Squamous cell carcinoma	60	M	3	2	2	1	0
E5	Squamous cell carcinoma	64	M	2	1	2	0	0
E6	Large cell carcinoma	55	M	0	1	2	0	0
E7	Small cell carcinoma	56	M	0	1	2	0	0
E8	Small cell carcinoma	43	M	0	1	2	0	0
F1	Adjacent Normal	60	M	-	-	-	-	-
F2	Adjacent Normal	44	M	-	-	-	-	-
F3	Interstitial pneumonia	54	M	-	-	-	-	-
F4	Interstitial pneumonia	60	M	-	-	-	-	-
F5	Adjacent Normal	64	M	-	-	-	-	-
F6	Adjacent Normal	55	M	-	-	-	-	-
F7	Adjacent Normal	56	M	-	-	-	-	-
F8	Interstitial pneumonia	43	M	-	-	-	-	-
-	Hepatocellular liver cancer (tissue marker)	55	M	-	-	-	-	-

Each core indicates the tissue type, patient´s age and sex, and tumor grade, stage, size, and metastases to lymph nodes and to distant sites.

**Table 3:** Complete description of TMA T041.

Core TMA3 T041	Histological Type	Age	Sex	Grade	Size	Lymph Node Metastases	Distant Metastases	Stage
A1	Adenocarcinoma	64	M	2	2	3	0	IIIb
A2	Adenocarcinoma	64	M	2	2	3	0	IIIb
A3	Squamous cell carcinoma	67	M	2–3	2	1	0	II
A4	Squamous cell carcinoma	67	M	2–3	2	1	0	II
A5	Adenocarcinoma	64	M	2	2	3	0	IIIb
A6	Adenocarcinoma	64	M	2	2	3	0	IIIb
A7	Squamous cell carcinoma	67	M	2–3	2	1	0	II
A8	Squamous cell carcinoma	67	M	2–3	2	1	0	II
B1	Large cell carcinoma	50	M	-	2	0	0	I
B2	Large cell carcinoma	50	M	-	2	0	0	I
B3	Small cell carcinoma	51	F	-	2	1	0	II
B4	Small cell carcinoma	51	F	-	2	1	0	II
B5	Large cell carcinoma	50	M	-	2	0	0	I
B6	Large cell carcinoma	50	M	-	2	0	0	I
B7	Small cell carcinoma	51	F	-	2	1	0	II
B8	Small cell carcinoma	51	F	-	2	1	0	II
C1	Normal lung tissue	21	F	-	-	-	-	-
C2	Normal lung tissue	21	F	-	-	-	-	-
C3	Normal lung tissue	21	F	-	-	-	-	-
C4	Normal lung tissue	21	F	-	-	-	-	-
C5	Normal lung tissue	21	F	-	-	-	-	-
C6	Normal lung tissue	21	F	-	-	-	-	-
C7	Normal lung tissue	21	F	-	-	-	-	-
C8	Normal lung tissue	21	F	-	-	-	-	-
-	Malignant melanoma (tissue marker)	58	M	-			-	

Each core indicates the tissue type, patient´s age and sex, and tumor grade, stage, size, and metastases to lymph nodes and to distant sites.

In the three TMAs we had a combined total of 58 patients and 36 controls (see Materials and methods for power analyses). Tumors were SCC (n = 24), AC (n = 20), SCLC (n = 8) and large cell carcinomas (LCC) (n = 6). The identity of the non-cancer controls is indicated in the tables. SCLC lack Rb (over 90%) due to the mutational inactivation of the *RB1* locus [32], therefore they are suitable internal negative controls included in the TMAs. The raw scoring data for all cores stained for phospho-Rb S249, phospho-Rb T821 and p39 in each TMA is shown in S2, S3 and S4 Tables. To account for tumor heterogeneity at least one pathologist scored the whole tumor area. As can be seen in Table 4, average staining scores for phospho-Rb-S249 and phospho-Rb-T821 did not significantly differ between cancer tissues relative to non-cancerous controls, even when breaking the scoring down into lung cancer sub-types. The biggest difference was observed for p39 staining, which showed a score of 2.4 in cancer tissues, relative to 0 for non-cancer controls.

**Table 4:** Averaged scores from three pathologists and the Aperio system, for phospho-Rb S249, phospho-Rb T821 and p39 staining, broken down by tissue type. In average, staining scores for phospho-Rb S249 and phospho-Rb T821 did not significantly differ between cancer tissues relative to non-cancerous controls, even when breaking the scoring down into histological subtypes. The difference of the biggest magnitude was the one observed for p39 staining, which showed a score of 2.4 in cancer tissues, relative to 0 for non-cancer controls.

***CANCER***	
***Tissue Type***	**Average Score**
*Squamous Cell Carcinoma (n = 24)*	
*Rb S249*	1.4
*Rb T821*	1.8
*p39*	1.7
*Adenocarcinomas (n = 20)*	
*Rb S249*	1.1
*Rb T821*	1.5
*p39*	3
*Small Cell Lung Carcinomas (n = 8)*	
*Rb S249*	1
*Rb T821*	2
*p39*	Not Aplicable
*Large Cell Carcinoma (n = 6)*	
*Rb S249*	1.5
*Rb T821*	1.5
*p39*	Not Aplicable
***Average for all Cancer Tissues (n = 58)***	
*Rb S249*	1.3
*Rb T821*	1.7
*p39*	2.4
***NON-CANCER***	
***Tissue Type***	**Average Score**
*Adjacent Normal Lung Tissue (n = 30)*	
*Rb S249*	0.4
*Rb T821*	1.5
*p39*	0
*Other*, *benign diseases (n = 6)*	
*Rb S249*	1.3
*Rb T821*	1.3
*p39*	0
***Average for all Non-Cancer Tissues (n = 36)***	
*Rb S249*	1.3
*Rb T821*	1.4
*p39*	0

**Fig 5:**
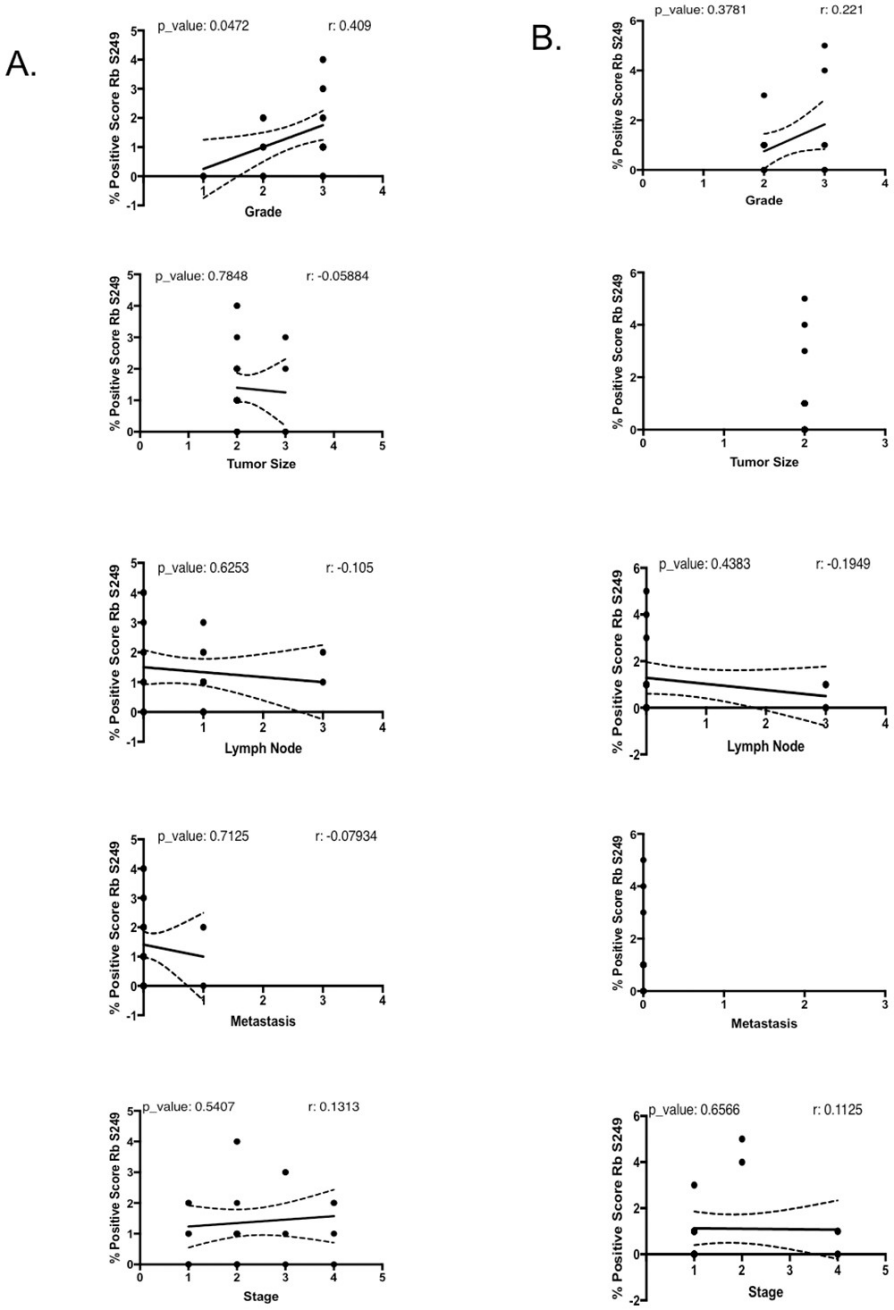
Rb S249 phosphorylation correlates with grading in SCC tumors. Correlation coefficient analyses to determine whether in the SCC subtype (A), the IHC score for Rb S249 phosphorylation correlates with any of the clinical parameters accompanying the tumor samples in the TMAs. In SCC, the IHC score for Rb S249 phosphorylation positively correlated only with tumor grade, with a correlation coefficient of 0.409, and a p-value of 0.0472. (B) In AC, Rb S249 phosphorylation did not correlate with any clinical parameter.

We next performed correlation analyses to determine if phospho-Rb-S249, phospho-Rb-T821, and p39 staining scores correlate with tumor grade, stage, size, lymph node invasion and metastases. Our analyses were limited to the AC and SCC subtypes since the low number of SCLC in our samples (n = 8) was not sufficient for statistical analyses. Fig 5 shows the correlation between phospho-Rb S249 staining scores and the various clinical parameters in SCC (Fig 5A) and AC (Fig 5B) tumors. In SCC, a high phospho-Rb S249 score was positively correlated with tumor grade (correlation coefficient of 0.409, p-value of 0.0472), meaning that the SCC tumors with higher grades are more likely to have the highest number of phospho-Rb S249 positive cells per 40X visual field. This correlation was exclusive to SCC since we did not find it in AC, where the phospho-Rb S249 score did not correlate with any of the clinical parameters.

**Fig 6:**
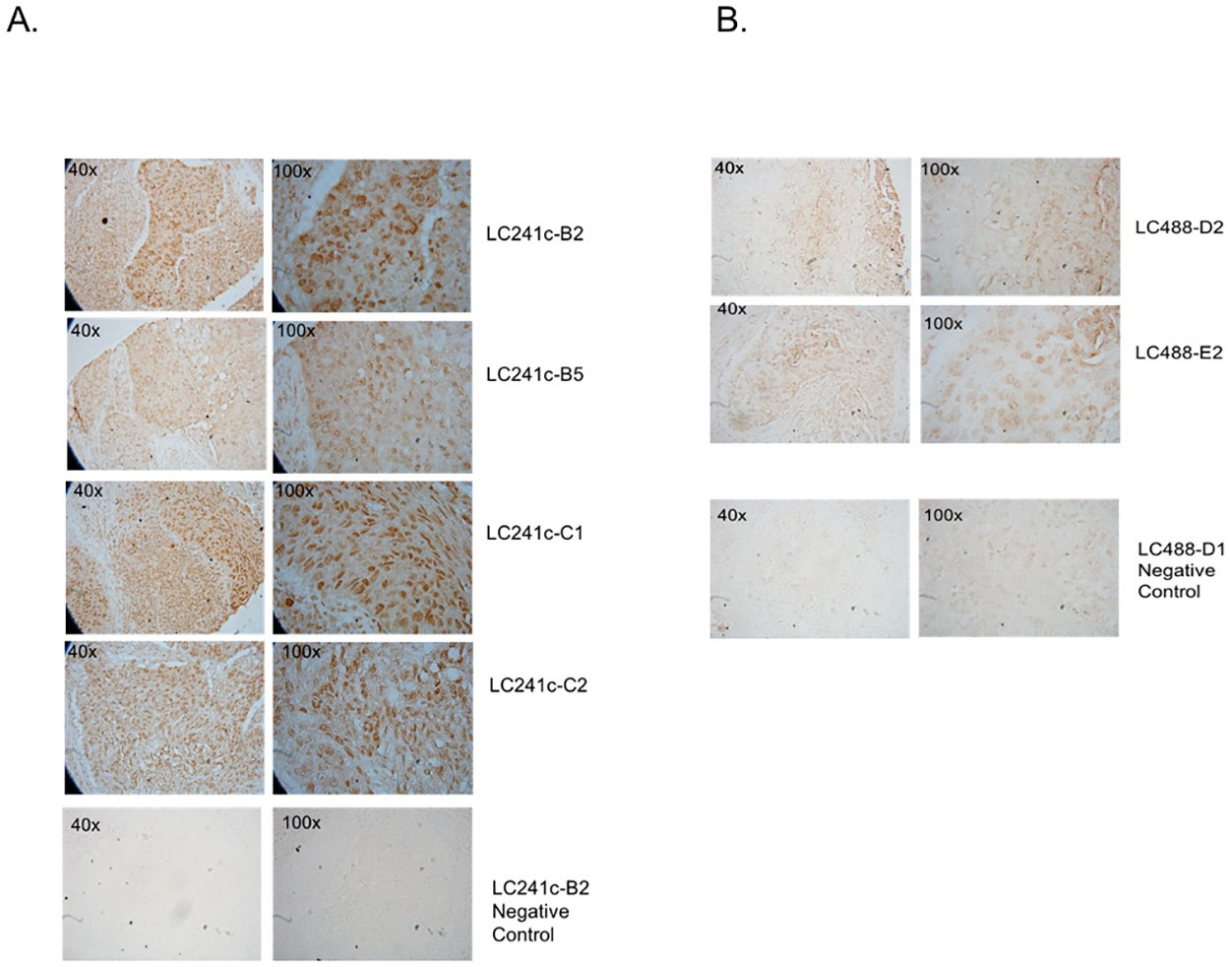
High grade tumors have strong staining for Rb S249 phosphorylation. IHC staining showing a strong nuclear staining for phospho-Rb S249 in four high grade SCC tissue cores (A). These four SCC cores all had a tumor grading of 3 and a Rb phospho-S249 staining score of 3. In contrast, grade 1 SCC cores had a Rb phospho-S249 staining score of 0 (B). Panels at the bottom of each figure are negative controls in which the primary antibody was omitted from the IHC protocol.

**Fig 7:**
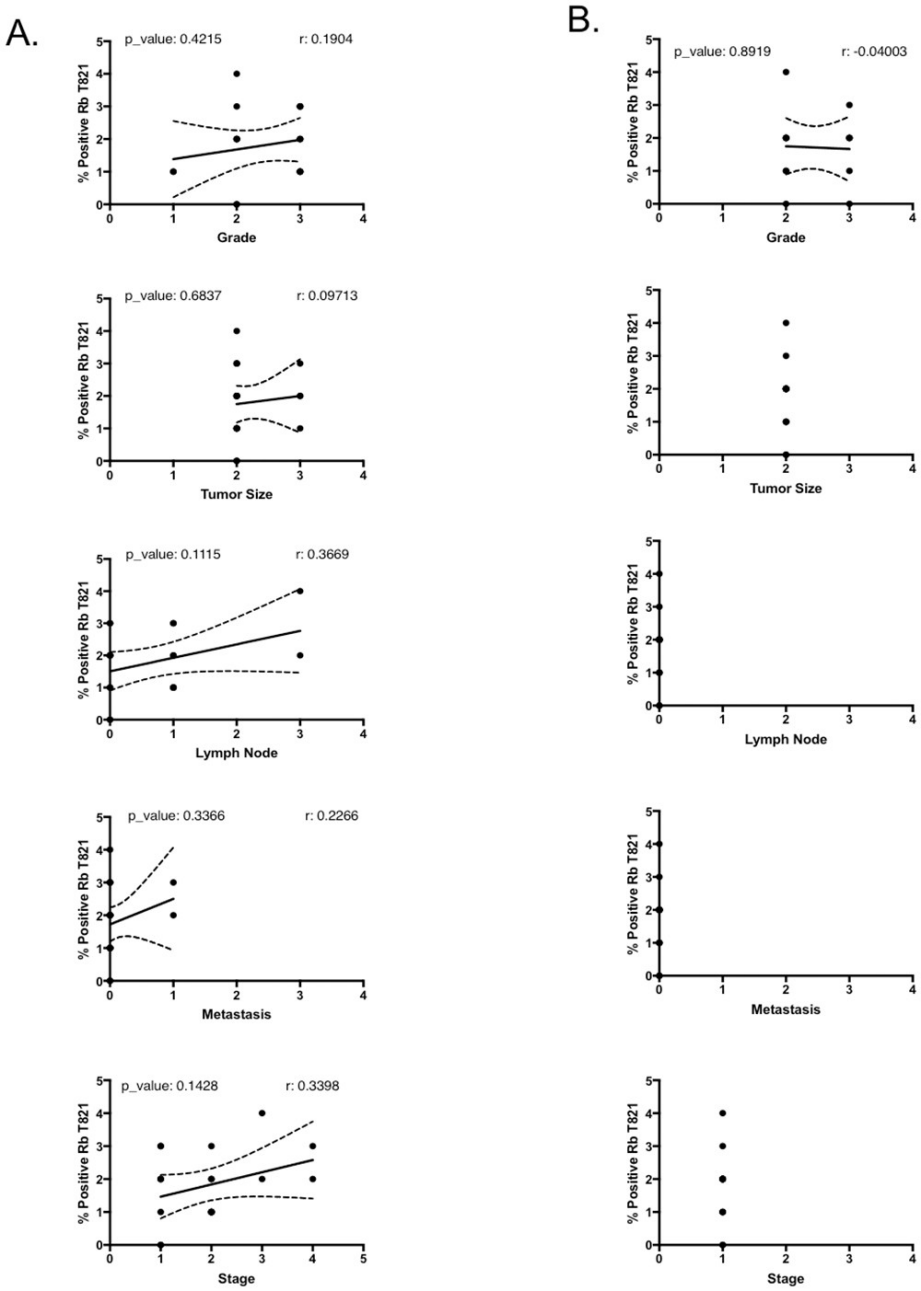
Rb T821 phosphorylation did not correlate with any of clinical parameter. Correlation coefficient analyses to determine whether in the SCC (A) or in the AC subtypes (B), the IHC score for Rb phosphorylation on T821 correlates with any of the clinical parameters accompanying the tumor samples in the TMAs.

Four out of four SCCs with strong phospho-Rb S249 staining (score >3) were grade 3 or higher tumors, while in AC, 2 out of 3 tumors with phospho-Rb S249 staining score >3 had a tumor grading of 3 or higher (see cores C1, C2, C5, C6, B2, B5 in TMA1 LC241c, and core B7 in TMA2 LC488 in S2 and S3 Tables). Fig 6A shows strong nuclear staining for Rb S249 phosphorylation in high tumor grade cores LC241c-B2, LC241c-B5, LC241c-C1 and LC241c-C2, all of which are SCC with a tumor grading of 3 and a Rb phospho-S249 staining score of 3. In contrast, Fig 6B shows grade 1 SCC cores LC488-D2 and LC488-E2 that have a Rb phospho-S249 staining score of 0. We also looked for correlations between phospho-Rb T821 staining scores and clinical parameters, but as can be seen in Fig 7, phospho-Rb T821 staining did not correlate with any of these parameters, neither in SCC (Fig 7A) nor in AC (Fig 7B).

**Fig 8:**
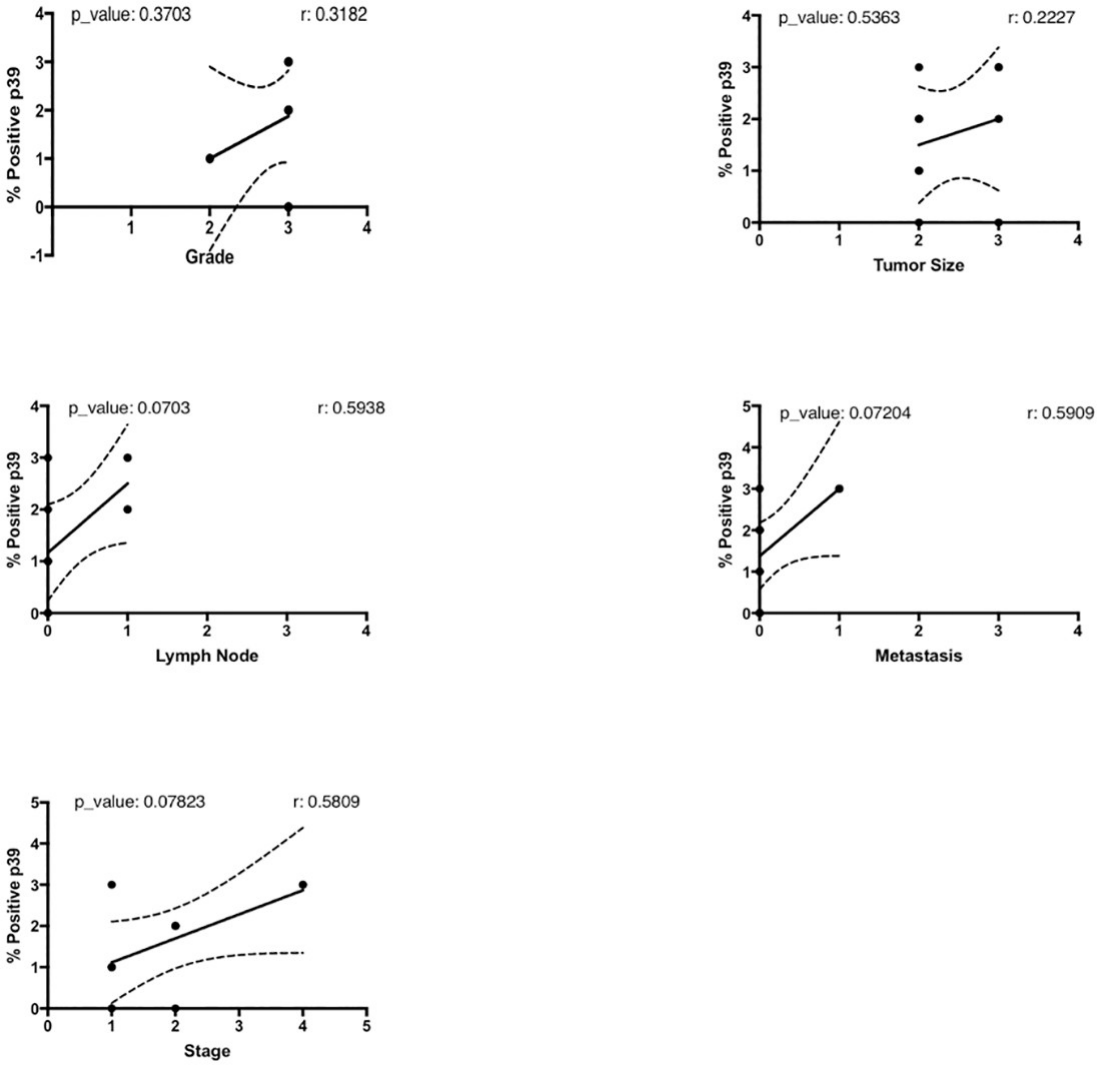
p39 staining correlates with staging and metastasis in SCC tumors. Correlation coefficient analyses to determine whether in SCC subtype, the IHC score for p39 staining correlates with any of the clinical parameters accompanying the tumor samples in the TMAs. In SCC, the IHC score for p39 staining positively correlated with metastasis to the lymph nodes (correlation coefficient = 0.5938, p-value = 0.0703), with tumor staging (correlation coefficient = 0.5809, p-value = 0.07823), and with metastasis to distant sites (correlation coefficient = 0.5909, p-value = 0.07204).

**Fig 9:**
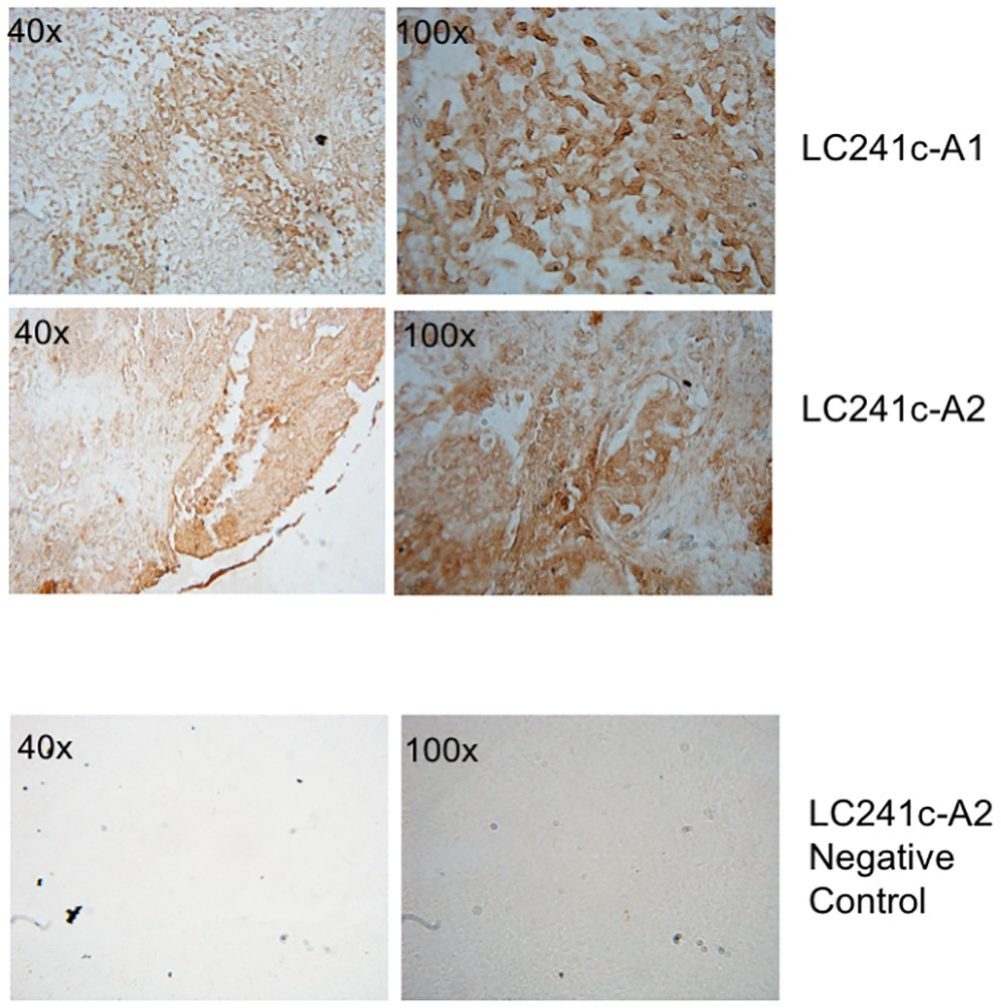
Strong p39 staining is observed in metastatic tumors. Strong p39 IHC staining of SCC tissue cores LC241c-A1 and LC241c-A2 from stage 4 patients with both lymph node and distant metastases. These cores have a p39 staining score of 3, a strong phospho-Rb S249 staining and a grade of 3. The two panels at the bottom illustrate negative controls (no primary antibody) of the LC241c-A2 core.

We also studied the relation between p39 staining scores and clinical parameters. In this case also the most significant correlations were found exclusively in SCC. In SCC tumor samples, p39 staining positively correlated with tumor staging (r = 0.58, p-value = 0.078), lymph node invasion (r = 0.59, p-value = 0.07), and distant metastases (r = 0.59, p-value = 0.07) (Fig 8). Among all our SCC samples, we only had two cores from patients that had both lymph node metastases and distant metastases (cores TMA1 LC241c A1 and A2 in S2 and S3 Tables), which also happen to be stage 4 (the highest we have amongst our samples), and these cores have a p39 staining score of 3. The strong IHC staining for p39 of these two high stage cores with lymph and distant metastases is shown in Fig 9. As can be seen in Table 2, all our AC samples were the same in stage, tumor size and spreading to lymph nodes and distant sites, therefore, we could not do correlation analyses between p39 expression and any of these parameters.

**Fig 10:**
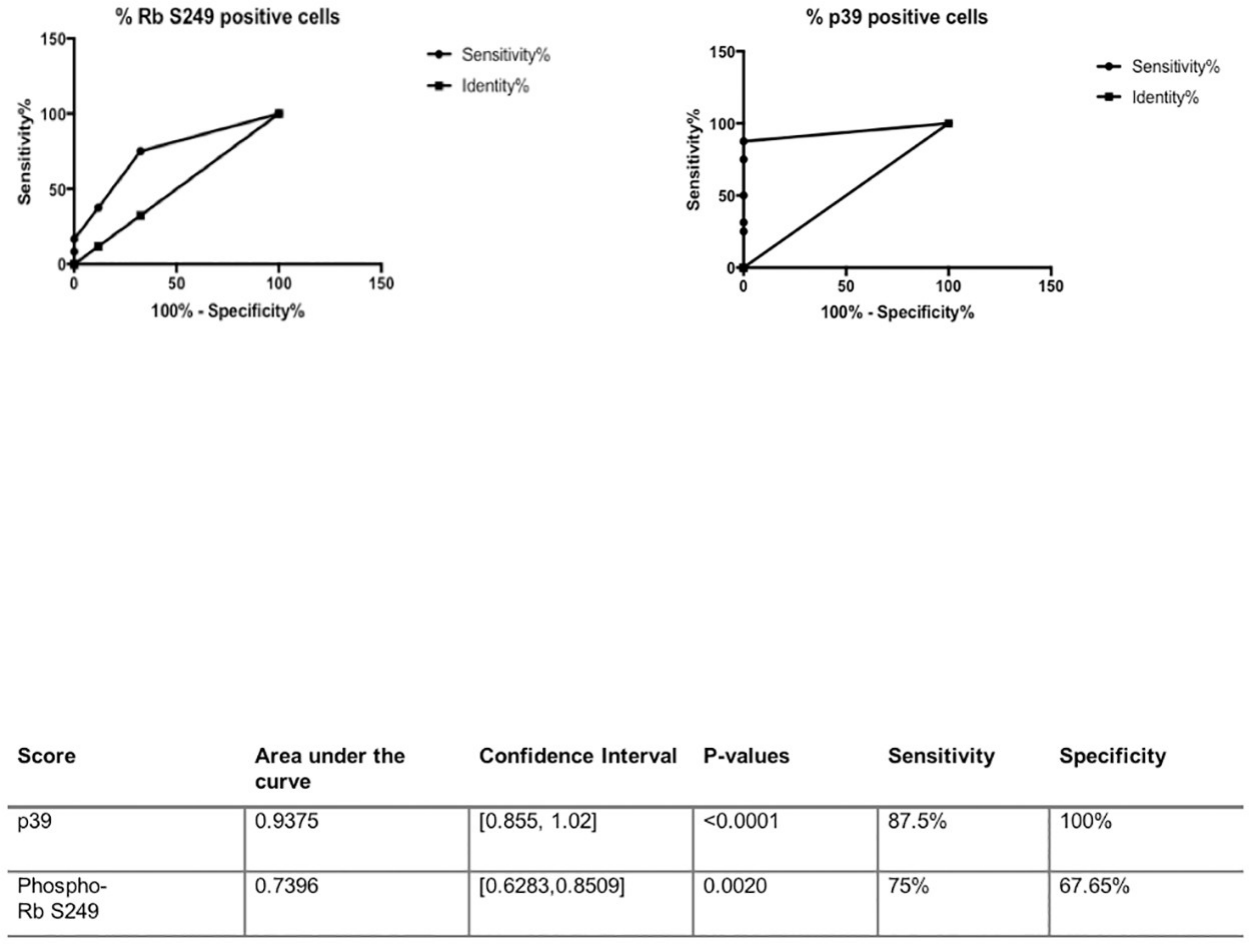
Receiver Operating Characteristic (ROC) curves for phospho-Rb S249 and p39, and the table showing the associated numerical values for area under the curve, confidence interval, p-value, sensitivity, and specificity. The area under the curve is a range between 0.5 (no predictive power) and 1.0 (perfect predictive accuracy). The phospho-Rb S249 score had an area under the curve of 0.7396, while the p39 score had an area of 0.9375, both being close to 1.0 and thus indicating a strong predictive value for grading and staging and metastasis, respectively. Phospho-Rb S249 and p39 scores had a sensitivity of 75% and 87.5%, respectively, meaning that 75% of tumors with high phospho-Rb S249 score also had high grading, and 87.5% of tumors with high p39 score also had high staging and lymph node and distant metastasis. Regarding specificity, the numbers for the phospho-Rb S249 and the p39 scores were 67.65% and 100%, respectively, meaning that 67.65% of the tumors with low scores for phospho-Rb S249 score were also of low grade, while none of the tumors negative for p39 were of a higher stage or had metastasis to lymph nodes or distant sites.

We next conducted a Receiver Operating Characteristic (ROC) analysis to confirm the diagnostic capabilities of phospho-Rb S249 regarding grading and p39 regarding staging and lymph nodes and distant metastasis. The top of Fig 10 shows the ROC curves for phospho-Rb S249 and p39 scores, and the table below shows the associated numerical values for the area under the curve, confidence interval, p-value, sensitivity, and specificity. The area under the curve is usually classified as a range between 0.5 (no predictive power) and 1.0 (perfect predictive accuracy). As can be seen in this table, phospho-Rb S249 score had an area under the curve of 0.7396, while p39 score had an area of 0.9375, both being close to 1.0 and thus indicating a strong predictive value for grading and staging and metastasis, respectively. Phospho-Rb S249 and p39 scores had a sensitivity of 75% and 87.5%, respectively, meaning that 75% of the tumor samples with high phospho-Rb S249 score also had high grading, and 87.5% of the tumor samples with high p39 score had high staging and lymph node involvement and distant metastasis. Regarding specificity, the numbers for the phospho-Rb S249 and the p39 scores were 67.65% and 100%, respectively, meaning that 67.65% of the tumor samples with low scores for phospho-Rb S249 score were also of low grade, while none of the tumor samples negative for p39 were of a higher stage or had metastasis to lymph nodes or distant sites.

Finally, we wanted to determine if the biomarkers of phospho-Rb S249 and phospho-Rb T821, E-cadherin, and p39 expression have a stronger predictive performance when considered together. We included E-cadherin in the analysis given its known prognostic value. Even when we did not find a correlation between Rb phosphorylation in T821 and any clinical parameter, we decided to test it in the linear regression model to check if it has predictive potential when combined with the other markers. We performed an ANOVA analysis that confirmed that Rb phosphorylation on S249, E-cadherin and p39 expression are indeed significantly associated, and thus these were used as variables to be entered into a linear regression model, with a p-value threshold set to 0.10. Consistent with our previous analyses, Rb phosphorylation in T821was not associated with SCC staging. Based on this, our regression model is represented by the following equation: Stage = 7.79–0.0669 X% score for Rb S249 positive cells—0.1432 X% score for p39 positive cells—0.1509 X% score for E-cadherin positive cells.

To confirm the association between p39, E-cadherin and phospho-Rb S249 scores, we randomly selected 70% of our SCC tumors from our TMAs and used our linear regression model to calculate tumor staging considering only the p39 score, only phospho-Rb S249 score, only E-cadherin and the three markers phospho-Rb S249, E-cadherin and p39 scores combined together. We then compared the tumor staging generated by our regression model with the staging information that accompanied each tumor core (provided by the vendor). We predicted that our linear regression model produces staging values that more closely resemble the staging provided by the vendor when we consider phospho-Rb S249, E-cadherin and p39 scored together, as opposed to each score individually.

Tables 5–9 shows, for 7 randomly picked SCC tumors, phospho-Rb S249, p39, and E-cadherin scores (*first three columns*, *respectively*), the staging information provided by the vendor (*Patient´s Stage*, *fourth column*), the stage predicted by our linear regression model considering only the phospho-Rb s249 score (*5th column in* Table 5), the stage predicted by our linear regression model considering only the p39 score (*5th column in* Table 6), the stage predicted only using E-cadherin score (*5*^*th*^ *column in* Table 7), and the stage predicted by our linear regression model considering the three biomarkers phospho-Rb S249, p39, and E-cadherin scores (*5th column in* Table 8). In these 4 tables, the rightmost columns labelled “Error” represent the differences between the vendor stage and our linear regression analysis stage, a value of 0 indicating that both stages are exactly the same, while a number other than 0 means that the two stages were different by a magnitude equal to the number indicated in the column. As can be seen in Table 5, phospho-Rb S249 score alone failed to provide the same staging as the staging data accompanying the tumor cores as provided by the vendor in all 7 cases. As shown in Table 6, p39 scoring by itself consistently overestimated the stage by a factor between 1 and 3 in 5 out of 7 samples. Likewise, as shown in Table 7, E-cadherin score by itself failed in 7 out of 7 instances to accurately match the stage clinical data provided by the vendor, in all cases greatly overestimating the staging. However, as shown in Table 8, the combination of phospho-Rb S249 and p39 scores was able to accurately match the staging clinical data provided by the vendor in 4 out of 7 instances, and in the two patients in which the staging did not match, the difference between stages was only of 1–2. Therefore, the predictive power of the model is increased when these two markers are considered together. If, in addition to phospho-Rb S249 and p39 scores, we add also the E-cadherin staining scores, then the predictive power of the model increases to accurately predict staging in 5 out 7 samples, with a difference of only 1 in the two mismatched samples (Table 9).

**Table 5:** A random sample of SCC tumors from our TMAs were selected to calculate tumor staging using our linear regression model when considering only phosphor-Rb S249 score. The tables show the score for phospho-Rb S249, p39, E-cadherin and the patient´s tumor stage (*first fourth columns*, *respectively*). The fifth column shows the stage predicted by our linear regression analysis only when considering the phospho-Rb S249 score. The Error (sixth) columns in all five tables show the difference between the vendor score and our linear regression analysis score, a value of 0 indicating identical scores, while a number other than 0 means that the two scores were different by a magnitude equal to the number indicated in the column. None of the 7 samples in Rb S249 score alone was able to predict the staging with an error of 0. Based on this analysis, Rb S249 score alone does not have enough precision to predict tumor staging accurately.

Phospho-Rb S249	p39	E-cadherin	Patient's Stage	Regression Model Stage: phospho Rb S249 only	Error
10	0	0	4	7	3
9	0	0	4	7	3
16	0	0	1	7	6
16	0	0	1	7	6
10	0	0	2	7	5
27	0	0	2	6	4
17	0	0	2	7	5
4	0	0	2	8	6

**Table 6:** A random sample of SCC tumors from our TMAs were selected to calculate tumor staging using our linear regression model when considering only p39 score. The tables show the score for phospho-Rb S249, p39, E-cadherin and the patient´s tumor stage (*first fourth columns*, *respectively*). The fifth column shows the stage predicted by our linear regression analysis only when considering the p39 score only. The Error (sixth) columns in all five tables show the difference between the vendor score and our linear regression analysis score, a value of 0 indicating identical scores, while a number other than 0 means that the two scores were different by a magnitude equal to the number indicated in the column. p39 alone was only able to accurately predict tumor stage in 2 out of 7 samples.

Phospho-Rb S249	p39	E-cadherin	Patient's Stage	Regression Model Stage:p39 only	Error
0	26	0	4	4	0
0	25	0	4	4	0
0	27	0	1	4	3
0	40	0	1	2	1
0	31	0	2	3	1
0	28	0	2	4	2
0	35	0	2	3	1

**Table 7:** A random sample of SCC tumors from our TMAs were selected to calculate tumor staging using our linear regression model when considering only E-cadherin score. The tables show the score for phospho-Rb S249, p39, E-cadherin and the patient´s tumor stage (*first fourth columns*, *respectively*). The fifth column shows the stage predicted by our linear regression analysis only when considering E-cadherin. The Error (sixth) columns in all five tables show the difference between the vendor score and our linear regression analysis score, a value of 0 indicating identical scores, while a number other than 0 means that the two scores were different by a magnitude equal to the number indicated in the column. E-cadherin was unable to accurately predict stage in 7 out of 7 samples.

Phospho- Rb S249	p39	E-cadherin	Patient's Stage	Regression Model Stage: E-cadherin only	Error
0	0	0	4	8	4
0	0	0	4	8	4
0	0	12	1	6	5
0	0	2	1	7	6
0	0	1	2	8	6
0	0	2	2	7	5
0	0	1	2	8	6

**Table 8:** A random sample of SCC tumors from our TMAs were selected to calculate tumor staging using our linear regression model when considering only E-cadherin score. The tables show the score for phospho-Rb S249, p39, E-cadherin and the patient´s tumor stage (*first fourth columns*, *respectively*). The fifth column shows the stage predicted by our linear regression analysis when considering phospho-Rb S249 and p39 scores together. The Error (sixth) columns in all five tables show the difference between the vendor score and our linear regression analysis score, a value of 0 indicating identical scores, while a number other than 0 means that the two scores were different by a magnitude equal to the number indicated in the column. The combined scores of phospho-Rb S249 and p39 accurately predicted the stage in 4 out of 7 samples.

Phospho-Rb S249	p39	E-cadherin	Patient's Stage	Regression Model Stage: phospho-Rb S249 and p39	Error
10	26	0	4	3	1
9	25	0	4	4	0
16	27	0	1	3	-2
16	40	0	1	1	0
10	31	0	2	3	-1
31	28	0	1	1	0
38	27	0	1	1	0

**Table 9:** A random sample of SCC tumors from our TMAs were selected to calculate tumor staging using our linear regression model when considering only E-cadherin score. The tables show the score for phospho-Rb S249, p39, E-cadherin and the patient´s tumor stage (*first fourth columns*, *respectively*). The fifth column shows the stage predicted by our linear regression analysis the when the three scores are combined. The Error (sixth) columns in all five tables show the difference between the vendor score and our linear regression analysis score, a value of 0 indicating identical scores, while a number other than 0 means that the two scores were different by a magnitude equal to the number indicated in the column. Finally, all three scores combined accurately predicted the tumor stage in 5 out of 7 samples. A linear regression model having only linear terms can result in positive or negative predictions if no other restrictions are present. In contrast, the three scores combined were able to accurately predict tumor stage in 5 out of 7 samples, and in the two samples in which the prediction was not perfectly accurate, they overestimated tumor stage by only a magnitude of 1. This suggests that the combination of phospho-Rb S249, p39, and E-cadherin scores may have strong predictive power for assessing stage.

Phospho-Rb S249	p39	E-cadherin	Patient's Stage	Regression Model Stage: All variables	Error
10	26	0	4	3	1
9	25	0	4	4	0
16	27	12	1	1	0
16	40	2	1	1	0
10	31	1	2	3	-1
31	28	3	1	1	0
38	27	0	1	1	0

In summary, our data show a correlation between phospho-Rb S249 scoring and tumor grading, and between p39 scoring and tumor staging and metastases to lymph nodes and to distant sites. Furthermore, our data show that when combined with the E-cadherin score, the combined phospho-Rb S249 and p39 scores can increase the accuracy of staging prediction.

## Discussion

### The relevance of the retinoblastoma phosphoprotein and p39 as lung cancer biomarkers

Here we describe our work aimed at identifying clinically informative biomarkers which could refine the current pathology workflows used in lung cancer diagnostics. Our data point in particular to p39 overexpression and Rb phosphorylation on serine 249 as biomarkers that could refine current methods used for NSCLC grading, histologic sub-classification and assessment of metastatic potential. Our main findings are that a) increased phosphorylation of Rb S249 positively correlates with grading only in SCC; b) increased Rb S249 phosphorylation is preserved in high grade SCC, therefore this biomarker could facilitate the sub-classification of poorly differentiated SCCs; c), increased expression of p39 in SCC correlates with staging and metastasis to lymph nodes and distant sites, and d), the combined scores for Rb S249 phosphorylation and p39 expression are stronger predictors of SCC staging than each of them separately.

There is a sound rationale behind Rb phosphorylation and p39 expression as potential lung cancer biomarkers. Oncogenic Rb hyper-phosphorylation is common in NSCLC [33], and being a strong driver of oncogenesis, this event may be a constant in a large fraction of tumor cells not subject to tumor heterogeneity. We and other have produced evidence showing that Rb induces cell adhesion [9–16], thus implicating Rb loss in cellular changes germane to tumor invasiveness and metastasis. Rb´s many functions are regulated by distinct phosphorylation codes [2,7,17–19,30,31,34–36][37–40], therefore we can postulate that a particular Rb phosphorylation could adversely affect cell adhesion and thus have metastasis-predicting potential. Finally, a technical, but nevertheless important aspect to consider is that the availability of high-quality Rb phospho-site specific antibodies makes it feasible to incorporate the IHC staining of phosphorylated Rb residues in the pathology staging and classification workflows.

Regarding p39, it is an activator of Cdk5, a kinase not strictly associated with cell cycle control but also involved in migration-associated cytoskeletal remodeling [41–43]. Cdk5 is reactivated in several metastatic human cancers, including lung, and is associated with poor prognosis [43–49]. Recent studies found Cdk5 protein up-regulation in 66 out of 95 (69.5%) NSCLC samples, and that Cdk5 expression correlated with decreased 5-year survival, poor differentiation, lymph node metastasis, and poor prognosis [44,45,48,49]. Cdk5 is essential for TGFβ1-induced EMT and breast cancer progression [47], further linking Cdk5 to aggressiveness and EMT. Rb phosphorylation by Cdk5 has been documented and Rb S249 and T821 residues are Cdk5 targets [38–42]. In spite of the evidence linking Cdk5 to metastasis, when we attempted its re-activation by forcing p39 expression in H1666 cells, we were not able to increase Rb S249 and T821 phosphorylation in them. Regardless, we still considered p39 as a potential biomarker in light of evidence arguing in favor of its clinical relevance (Fig 4E and 4F, S1 Fig, S1 Table).

### Phosphorylation of Rb S249 as a squamous cell carcinoma-specific histologic sub-classification biomarker

There is an urgent need for the identification of clinically informative biomarkers to improve several aspects of the lung cancer diagnosis workflow. While distinguishing between the NSCLC and SCLC major lung cancer subtypes with high accuracy may be relatively straightforward [50–62], there is still need for improvement regarding the histological distinction and sub-classification of NSCLC sub-types. NSCLC classification becomes progressively more difficult as tumors lose differentiation, and this challenge needs a resolution as NSCLCs comprise a clinically, pathologically and molecularly heterogeneous group of tumors with different treatment courses and clinical outcomes [63–66]. Therefore, there is the need to further sub-classify NSCLC into SCC, AC and LCC [63–66], and thus we postulate that Rb phosphorylation on S249, by being prominent in high-grade tumors exclusively of the SCC sub-type, could help to distinguish poorly differentiated SCC from other histological subtypes. When tumors are sufficiently differentiated, distinguishing between low-grade AC and SCC with high accuracy can be achieved by assessing tumor morphological features (WHO criteria for SCC sub-classification include the degree of keratinization and/or the presence of intercellular bridges, and squamous pearl formation, while AC is characterized by glandular differentiation and/or mucin production), in combination with immunohistochemical staining for tumor sub-type markers [63,65,67–82]. However, even when using IHC-detected molecular markers, further histological sub-classification, becomes progressively more difficult as the grade and therefore the degree of anaplasia increases, making the morphological and molecular features distinctive of either AC or SCC more difficult to discern [83]. Poorly differentiated SCC rarely express SCC markers, making high-grade SCC difficult to classify [72]. For example, highly differentiated AC and SCC can be readily distinguished on the basis of their cytokeratin (CK) expression profile; while CK5/6, CK14 and CK17 are predominantly expressed in SCC, CK7 and CK18 expression predominate in AC [84]. However, the expression of these markers has been shown to inversely correlate with grading and therefore high-grade tumors become difficult to classify due to anaplasia-associated CK down-regulation [84]. Furthermore, some IHC-detectable markers may not be 100% specific nor sensitive [63,67,72]. p63 preferentially stains SCC but may also stain AC and LCC, in one study up to 30% of ACs and 37% of LCC being p63 positive[85], meaning that p63 is not highly effective in discriminating between AC and SCC. TTF1 has a sensitivity of 70%-80% in ACs meaning that it fails to detect between 20–30% of AC [67,83,84,86–88]. Although CK7 expression is predominantly found in ACs, some studies have found approximately 8% of SCC testing positive for CK7 expression[84]. In another study, ACs were shown to have significant immunoreactivity for SCC markers, as 32% and 18% of them tested positive for the SCC markers p63 and CK5/6, respectively [67]. All things considered, even when employing an IHC panel, a fraction of poorly differentiated NSCLC still remains extremely difficult to subclassify as either AC or SCC, and therefore are given the designation of NSCLC-Not Otherwise Specified (NSCLC-NOS). NSCLC-NOS diagnostic rates increase when evaluating poorly differentiated tumors, and poor tumor differentiation has been identified as a predominant factor driving the increase in NSCLC-NOS diagnoses [89]. Therefore, even when using IHC, a fraction of NSCLC-NOS equivocal diagnoses still persists. For example, in a recent study with 74 tumors that were initially classified as NSCLC-NOS based purely on morphological criteria, after subjecting them to IHC staining for histology-specific antigens, 34 of them (46%) still remained as unclassified due to poor differentiation and lack of a specific immune-profile [70]. Up to the present, p63 is the SCC marker whose expression is the most resilient in the context of SCC anaplasia [67,88], but this advantage is offset by its poor specificity since as described above, some studies identified p63 staining in a large fraction of ACs [67]. Based on our data, and relevant to this issue, we would like to suggest that staining for Rb S249 phosphorylation in tumor biopsies could be added to the pathology workflow to help minimize the NSCLC-NOS classification when dealing with poorly differentiated tumors. An additional aspect that makes Rb S249 phosphorylation an attractive biomarker for undifferentiated SCC tumors is that the localization of its staining is predominantly nuclear. It has been previously suggested that nuclear markers should be employed as much as possible since these are more easily assessed in small specimens, even when the tissue suffers from sampling artifacts or shows poor cellularity due to necrosis [68–70,90]. Interpretation of nuclear staining is often less equivocal than staining of cytoplasmic markers like the currently used ones for SCC versus AC sub-classification [67].

### Combined phosphorylation of Rb S249 and p39 protein expression as a squamous cell carcinoma-specific staging biomarker

The prognosis for lung cancer patients is poor, with five-year survival rates for SCLC and NSCLC being 6% and 18%, respectively [63,91–94]. This poor prognosis may be explained by a proclivity for early metastasis, therefore the urgency for the identification of metastasis-predicting biomarkers that could be assessed in biopsies of primary tumors. The most precise predictors of metastasis and recurrence of NSCLC rely on post-resection assessment of tumor histology, this is called the pathological staging which is the closest to the true staging and is established by the surgeon and the pathologist during surgical resection of tumors including an accurate assessment of potential areas of spread, such as lymph nodes [95]. Unfortunately, approximately 85% of NSCLC patients are diagnosed with unresectable disease, meaning that pathological staging is not an option for them and therefore small biopsy specimens, sometimes only of the primary tumor site, are the primary material for diagnosis [62,63,67,70,83]. When resection is not an option, reliable staging strongly relies on imaging tests supplemented with multiple invasive procedures such as mediastinoscopy that impose considerable demands on patients whose health is significantly deteriorated at the moment of diagnosis, and therefore are unable or unwilling to subject to invasive testing. Hence the need for making biopsies of primary tumors as clinically informative as possible regarding metastatic potential. Identification of the proper combination of biomarkers to obtain the maximal diagnostic yield from small specimens, in particular by providing information about metastatic probability, is thus a priority for lung cancer diagnosis. In this work we have demonstrated that p39 levels in SCC tumors combined with Rb phosphorylation in S249 correlated with increased staging and with metastasis to lymph nodes and distant sites. Therefore, the combined staining of SCC tumor samples for Rb S249 phosphorylation and p39 expression, combined also with E-cadherin staining, could be added to the diagnostic workflow with the aim of assessing metastatic potential of the primary tumor.

### Concluding remarks

In summary, we have described a biomarker that could be useful in predicting SCC metastatic probability and grading. We originally identified this biomarker in the NSCLC cell line H520, which exhibits traits of epithelial-to-mesenchymal transition, relative to the NSCLC cell line H1666 which lacks such traits. It is worth noticing that H520 cells not only express EMT traits, but they are derived from a SCC tumor, in contrast to H1666 cells which are derived from AC. Therefore, the biomarker we identified, not only can be informative of tumor grade and metastatic potential but is also a SCC-specific marker that, importantly, shows resilience in the context of differentiation loss and can therefore serve to distinguish poorly differentiated SCC from AC. The fact that we identified the strongest associations between Rb S249 phosphorylation and p39 overexpression with tumor stage and grade only in SCC is fully consistent with the fact that we originally identified this biomarker in an EMT-positive SCC cell line. We believe this finding is highly significant, not only for its potential for being clinically informative, but also because currently there is a scarcity of prognostic signatures for SCC patients, the majority of lung cancer prognosis signatures identified to date being for the AC subtype [96,97].

## Supporting information

S1 Table NSCLC patients whose AC tumors show a TGFβ-induced EMT gene expression signature also show high p39 expression. The TGFβ-EMT signature was used to separate TMA5 into EMT-positive and EMT-negative patient populations. A t-test showed a statistically significant difference in p39 expression between patients with the TGFβ-EMT signature and those without the signature, with patients that are EMT-positive having higher p39 expression. CDK5R1 is the Cdk5 activator p35, while CDKR2 is the Cdk5 activator p39.
(DOCX)

S2 Table Detailed description of the TMA LC241C, including histological tumor type, average scoring for phospho-Rb S249, phospho-Rb T821 and p39, and the accompanying clinical data for each patient. The scores were averaged from the independent scores of 3 pathologists and the Aperio system.
(DOCX)

S3 Table Detailed description of the TMA LC488, including histological tumor type, average scoring for phospho-Rb S249, phospho-Rb T821 and p39, and the accompanying clinical data for each patient. The scores were averaged from the independent scores of 3 pathologists and the Aperio system.
(DOCX)

S4 Table Detailed description of the TMA T041, including histological tumor type, average scoring for phospho-Rb S249 and the accompanying clinical data for each patient. (DOCX)

S1 Fig The TGFβ-induced EMT signature is capable of segregating patients based on metastasis free survival, overall survival and time to metastasis in a tissue microarray cohort of 150 NSCLC patients (TMA5). The TGFβ-EMT signature was used to separate TMA5 into EMT-positive and EMT-negative patient populations. Using the EMT-positive population we then verified CDK5R2 (p39) expression on the different populations. (A) In TMA5, we studied the AC patients to observe if p39 expression correlated with the TGFβ-EMT signature. As p39 expression increases, more genes in the TGFβ-EMT are involved. (B) We also evaluated the metastatic patients in TMA5 to determine if p39 expression correlates with the TGFβ-EMT signature. As p39 expression increases in stage M1 more genes in the TGFβ-EMT are involved. Statistical analysis performed was Spearman correlation.
(TIF)

## Abbreviations

AC: adenocarcinoma
EMT: epithelial-to-mesenchymal transition
FAK: focal adhesion kinase
LCC: large cell carcinoma
NSCLC: non-small cell lung carcinoma
NSCLC-NOS: non-small cell lung carcinoma not-otherwise specified
Rb: retinoblastoma tumor suppressor
SCC: squamous cell carcinoma
SCLC: small cell lung carcinoma
TMA: tumor microarray
