# Supplementary material for: Hyper-phosphorylation of Rb S249 together with CDK5R2/p39 overexpression are associated with impaired cell adhesion and epithelial-to-mesenchymal transition: Implications as a potential lung cancer grading and staging biomarker
Source: PLoS One. 2018 Nov 19;13(11):e0207483. doi: 10.1371/journal.pone.0207483 (PMC6242691; doi:10.1371/journal.pone.0207483)
Supplement: S1 Table — The TGFβ-EMT signature was used to separate TMA5 into EMT-positive and EMT-negative patient populations. A t-test showed a statistically significant difference in p39 expression between patients with the TGFβ-EMT signature and those without the signature, with patients that are EMT-positive having higher p39 expression. CDK5R1 is the Cdk5 activator p35, while CDKR2 is the Cdk5 activator p39. (DOCX) [file pone.0207483.s001.docx]

| Gene | Mean TGFβ-EMT positive (n=99) | Mean TGFβ-EMT negative (n=104) | Difference in Mean | P-values |
| --- | --- | --- | --- | --- |
| CDK5 | 8.4688 | 8.4655 | 0.0033 | 0.9670 |
| CDK5R1 (p35) | 7.4336 | 6.3120 | 1.1216 | 1.36E-13 |
| CDK5R2 (p39) | 3.2951 | 1.8860 | 1.4091 | 2.31E-05 |
