# Supplementary material for: Hyper-phosphorylation of Rb S249 together with CDK5R2/p39 overexpression are associated with impaired cell adhesion and epithelial-to-mesenchymal transition: Implications as a potential lung cancer grading and staging biomarker
Source: PLoS One. 2018 Nov 19;13(11):e0207483. doi: 10.1371/journal.pone.0207483 (PMC6242691; doi:10.1371/journal.pone.0207483)
Supplement: S2 Table — The scores were averaged from the independent scores of 3 pathologists and the Aperio system. (DOCX) [file pone.0207483.s002.docx]

| Core TMA1 LC241C | Histological Type | Rb S249 | Rb T821 | p39 | Grade | Stage | Size | Lymph Node Metastases | Distant Metastases |
| --- | --- | --- | --- | --- | --- | --- | --- | --- | --- |
| A1 | Squamous Cell Carcinoma | 0 | 3 | 3 | 3 | 4 | 3 | 1 | 1 |
| A2 | Squamous Cell Carcinoma | 2 | 2 | 3 | 3 | 4 | 3 | 1 | 1 |
| A3 | Adjacent Normal | 0 | 2 | 0 | - | - | - | - | - |
| A4 | Adjacent Normal | 0 | 3 | 0 | - | - | - | - | - |
| A5 | Squamous Cell Carcinoma | 2 | 3 | 1 | 2 | 1 | 2 | 0 | 0 |
| A6 | Squamous Cell Carcinoma | 2 | 2 | 1 | 2 | 1 | 2 | 0 | 0 |
| A7 | Pulmonary edema with congestion | 2 | 2 | 0 | - | - | - | - | - |
| A8 | Pulmonary edema with congestion | 2 | 2 | 0 | - | - | - | - | - |
| B1 | Squamous Cell Carcinoma | 0 | 2 | 0 | 3 | 2 | 3 | 0 | 0 |
| B2 | Squamous Cell Carcinoma | 3 | 1 | 2 | 3 | 2 | 3 | 0 | 0 |
| B3 | Adjacent Normal | 2 | 1 | 0 | - | - | - | - | - |
| B4 | Adjacent Normal | 2 | 2 | 0 | - | - | - | - | - |
| B5 | Squamous Cell Carcinoma | 3 | 3 | 2 | 3 | 2 | 2 | 1 | 0 |
| B6 | Squamous Cell Carcinoma | 1 | 1 | 2 | 3 | 2 | 2 | 1 | 0 |
| B7 | Adjacent Normal | 2 | 2 | 0 | - | - | - | - | - |
| B8 | Adjacent Normal | 2 | 3 | 0 | - | - | - | - | - |
| C1 | Squamous Cell Carcinoma | 3 | 3 | 0 | 3 | 1 | 2 | 1 | 0 |
| C2 | Squamous Cell Carcinoma | 3 | 1 | 3 | 3 | 1 | 2 | 0 | 0 |
| C3 | Adjacent Normal | 2 | 1 | 0 | - | - | - | - | - |
| C4 | Adjacent Normal | 2 | 2 | 0 | - | - | - | - | - |
| C5 | Adenocarcinoma | 4 | 3 | 3 | 3 | 1 | 2 | 0 | 0 |
| C6 | Adenocarcinoma | 3 | 2 | 3 | 3 | 1 | 2 | 0 | 0 |
| C7 | Adjacent Normal | 2 | 2 | 0 | - | - | - | - | - |
| C8 | Adjacent Normal | 1 | 3 | 0 | - | - | - | - | - |
| - | Adrenal Gland Pheochromocytoma (tissue marker) | 42 | M | - | - | - | - | - | - |
