# Supplementary material for: Hyper-phosphorylation of Rb S249 together with CDK5R2/p39 overexpression are associated with impaired cell adhesion and epithelial-to-mesenchymal transition: Implications as a potential lung cancer grading and staging biomarker
Source: PLoS One. 2018 Nov 19;13(11):e0207483. doi: 10.1371/journal.pone.0207483 (PMC6242691; doi:10.1371/journal.pone.0207483)
Supplement: S3 Table — The scores were averaged from the independent scores of 3 pathologists and the Aperio system. (DOCX) [file pone.0207483.s003.docx]

| Core TMA2 LC488 | Histological Type | Rb S249 | Rb T821 | p39 | Grade | Stage | Size | Lymph Node Metastases | Distant Metastases |
| --- | --- | --- | --- | --- | --- | --- | --- | --- | --- |
| A1 | Adenocarcinoma | 0 | 0 | NS | 2 | 1 | 1 | 0 | 0 |
| A2 | Adenocarcinoma | 0 | 0 | NS | 2 | 1 | 2 | 0 | 0 |
| A3 | Adenocarcinoma | 0 | 2 | NS | 2 | 1 | 2 | 0 | 0 |
| A4 | Adenocarcinoma | 0 | 2 | NS | 3 | 1 | 2 | 0 | 0 |
| A5 | Adenocarcinoma | 1 | 1 | NS | 2 | 1 | 2 | 0 | 0 |
| A6 | Adenocarcinoma | 1 | 1 | NS | 2 | 1 | 2 | 0 | 0 |
| A7 | Adenocarcinoma | 1 | 1 | NS | 2 | 1 | 2 | 0 | 0 |
| A8 | Adenocarcinoma | 0 | 0 | NS | 3 | 1 | 2 | 0 | 0 |
| B1 | Adenocarcinoma | 0 | 0 | NS | 2 | 1 | 2 | 0 | 0 |
| B2 | Adenocarcinoma | 0 | 4 | NS | 2 | 1 | 2 | 0 | 0 |
| B3 | Adenocarcinoma | 1 | 2 | NS | 2 | 1 | 2 | 0 | 0 |
| B4 | Adenocarcinoma | 1 | 2 | NS | 3 | 1 | 2 | 0 | 0 |
| B5 | Adenocarcinoma | 1 | 2 | NS | 2 | 1 | 2 | 0 | 0 |
| B6 | Adenocarcinoma | 1 | 1 | NS | 2 | 1 | 2 | 0 | 0 |
| B7 | Adenocarcinoma | 3 | 2 | NS | 2 | 1 | 2 | 0 | 0 |
| B8 | Adenocarcinoma | 1 | 1 | NS | 3 | 1 | 2 | 0 | 0 |
| C1 | Adjacent Normal | 1 | 1 | NS | - | - | - | - | - |
| C2 | Adjacent Normal | 1 | 1 | NS | - | - | - | - | - |
| C3 | Adjacent Normal | 1 | 1 | NS | - | - | - | - | - |
| C4 | Adjacent Normal | 1 | 1 | NS | - | - | - | - | - |
| C5 | Adjacent Normal | 1 | 1 | NS | - | - | - | - | - |
| C6 | Adjacent Normal | 1 | 1 | NS | - | - | - | - | - |
| C7 | Stromal hyperplasia with congestion and edema | 1 | 1 | NS | - | - | - | - | - |
| C8 | Adjacent Normal | 1 | 1 | NS | - | - | - | - | - |
| D1 | Squamous cell carcinoma | 0 | 0 | NS | 2 | 1 | 2 | 0 | 0 |
| D2 | Squamous cell carcinoma | 0 | 1 | NS | 1 | 2 | 2 | 1 | 0 |
| Core TMA2 LC488 | Histological Type | Rb S249 | Rb T821 | p39 | Grade | Stage | Size | Lymph Node Metastases | Distant Metastases |
| D3 | Squamous cell carcinoma | 1 | 4 | NS | 2 | 3 | 2 | 3 | 0 |
| D4 | Squamous cell carcinoma | 1 | 2 | NS | 3 | 2 | 2 | 3 | 0 |
| D5 | Squamous cell carcinoma | 1 | 2 | NS | 2 | 1 | 2 | 0 | 0 |
| D6 | Large cell carcinoma | 2 | 1 | NS | 0 | 1 | 2 | 0 | 0 |
| D7 | Small cell carcinoma | 1 | 2 | NS | 0 | 1 | 2 | 0 | 0 |
| D8 | Small cell carcinoma | 1 | 2 | NS | 0 | 1 | 2 | 0 | 0 |
| E1 | Squamous cell carcinoma | 0 | 0 | NS | 2 | 1 | 2 | 0 | 0 |
| E2 | Squamous cell carcinoma | 0 | 1 | NS | 1 | 2 | 2 | 1 | 0 |
| E3 | Squamous cell carcinoma | 2 | 2 | NS | 2 | 3 | 2 | 3 | 0 |
| E4 | Squamous cell carcinoma | 1 | 1 | NS | 3 | 2 | 2 | 1 | 0 |
| E5 | Squamous cell carcinoma | 1 | 2 | NS | 2 | 1 | 2 | 0 | 0 |
| E6 | Large cell carcinoma | 1 | 2 | NS | 0 | 1 | 2 | 0 | 0 |
| E7 | Small cell carcinoma | 1 | 2 | NS | 0 | 1 | 2 | 0 | 0 |
| E8 | Small cell carcinoma | 1 | 2 | NS | 0 | 1 | 2 | 0 | 0 |
| F1 | Adjacent Normal | 0 | 1 | NS | - | - | - | - | - |
| F2 | Adjacent Normal | 1 | 1 | NS | - | - | - | - | - |
| F3 | Interstitial pneumonia | 1 | 1 | NS | - | - | - | - | - |
| F4 | Interstitial pneumonia | 1 | 1 | NS | - | - | - | - | - |
| F5 | Adjacent Normal | 1 | 1 | NS | - | - | - | - | - |
| F6 | Adjacent Normal | 1 | 1 | NS | - | - | - | - | - |
| F7 | Adjacent Normal | 1 | 1 | NS | - | - | - | - | - |
| F8 | Interstitial pneumonia | 1 | 1 | NS | - | - | - | - | - |
| - | Hepatocellular liver cancer (tissue marker) | - | - | NS | - | - | - | - | - |
