# Supplementary material for: Hyper-phosphorylation of Rb S249 together with CDK5R2/p39 overexpression are associated with impaired cell adhesion and epithelial-to-mesenchymal transition: Implications as a potential lung cancer grading and staging biomarker
Source: PLoS One. 2018 Nov 19;13(11):e0207483. doi: 10.1371/journal.pone.0207483 (PMC6242691; doi:10.1371/journal.pone.0207483)
Supplement: S4 Table — (DOCX) [file pone.0207483.s004.docx]

| Core TMA3 T041 | Histological Type | pRb S249 | Grade | Stage | Size | Lymph Node Metastases | Distant Metastases |
| --- | --- | --- | --- | --- | --- | --- | --- |
| A1 | Adenocarcinoma | 1 | 2 | IIIb | 2 | 3 | 0 |
| A2 | Adenocarcinoma | 0 | 2 | IIIb | 2 | 3 | 0 |
| A3 | Squamous cell carcinoma | 1 | 2--3 | II | 2 | 1 | 0 |
| A4 | Squamous cell carcinoma | 2 | 2--3 | II | 2 | 1 | 0 |
| A5 | Adenocarcinoma | 1 | 2 | IIIb | 2 | 3 | 0 |
| A6 | Adenocarcinoma | 0 | 2 | IIIb | 2 | 3 | 0 |
| A7 | Squamous cell carcinoma | 1 | 2--3 | II | 2 | 1 | 0 |
| A8 | Squamous cell carcinoma | 1 | 2--3 | II | 2 | 1 | 0 |
| B1 | Large cell carcinoma | 1 | - | I | 2 | 0 | 0 |
| B2 | Large cell carcinoma | 1 | - | I | 2 | 0 | 0 |
| B3 | Small cell carcinoma | 0 | - | II | 2 | 1 | 0 |
| B4 | Small cell carcinoma | 1 | - | II | 2 | 1 | 0 |
| B5 | Large cell carcinoma | 1 | - | I | 2 | 0 | 0 |
| B6 | Large cell carcinoma | 1 | - | I | 2 | 0 | 0 |
| B7 | Small cell carcinoma | 0 | - | II | 2 | 1 | 0 |
| B8 | Small cell carcinoma | 0 | - | II | 2 | 1 | 0 |
| C1 | Normal lung tissue | 1 | - | - | - | - | - |
| C2 | Normal lung tissue | 1 | - | - | - | - | - |
| C3 | Normal lung tissue | 0 | - | - | - | - | - |
| C4 | Normal lung tissue | 1 | - | - | - | - | - |
| C5 | Normal lung tissue | 1 | - | - | - | - | - |
| C6 | Normal lung tissue | 0 | - | - | - | - | - |
| C7 | Normal lung tissue | 0 | - | - | - | - | - |
| C8 | Normal lung tissue | 0 | - | - | - | - | - |
| - | Malignant melanoma (tissue marker) | - | - |  | - | - | - |
