# Supplementary figures and images for: Hyper-phosphorylation of Rb S249 together with CDK5R2/p39 overexpression are associated with impaired cell adhesion and epithelial-to-mesenchymal transition: Implications as a potential lung cancer grading and staging biomarker
Source: PLoS One. 2018 Nov 19;13(11):e0207483. doi: 10.1371/journal.pone.0207483 (PMC6242691; doi:10.1371/journal.pone.0207483)

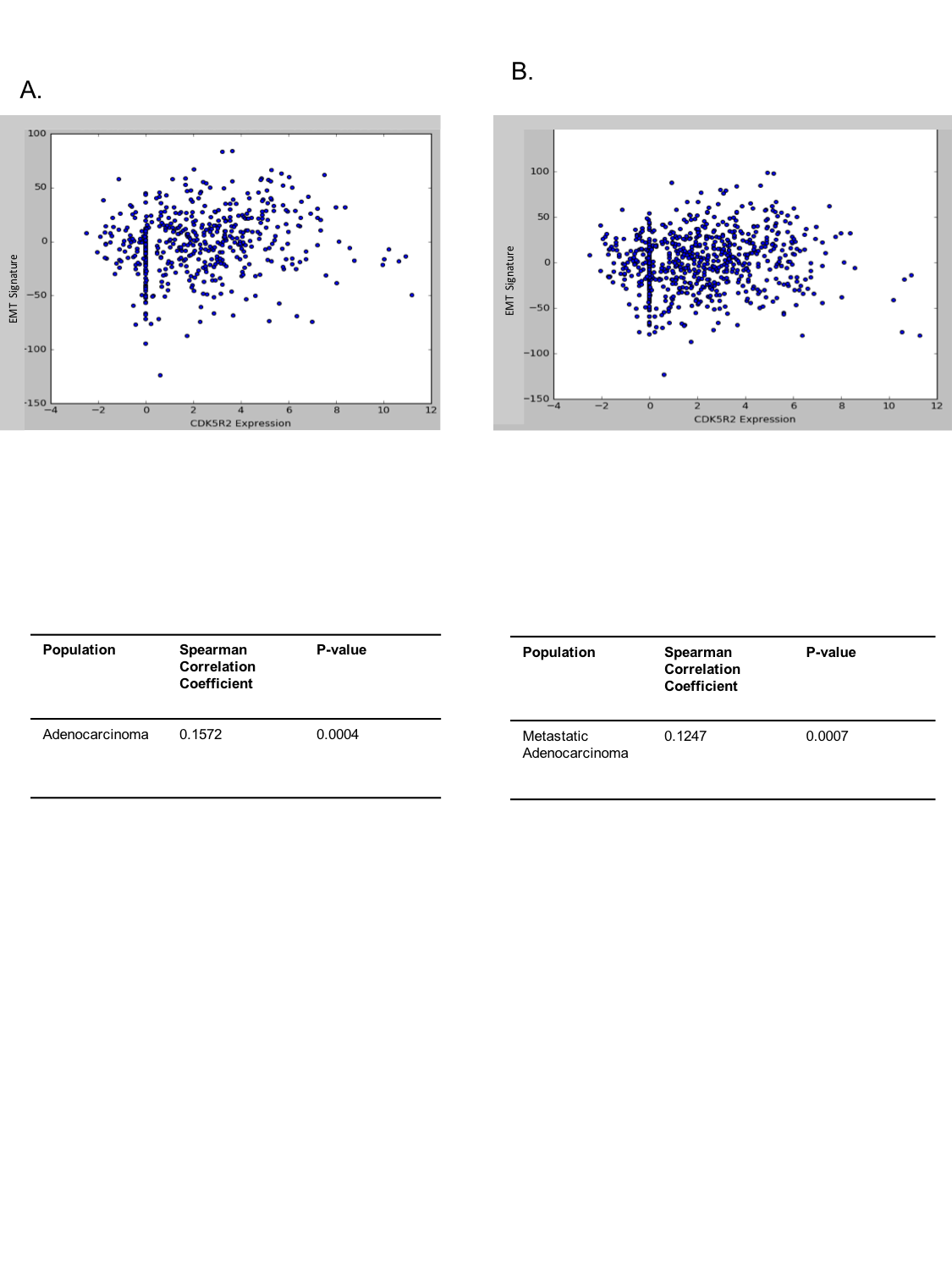

Supplement: S1 Fig — The TGFβ-EMT signature was used to separate TMA5 into EMT-positive and EMT-negative patient populations. Using the EMT-positive population we then verified CDK5R2 (p39) expression on the different populations. (A) In TMA5, we studied the AC patients to observe if p39 expression correlated with the TGFβ-EMT signature. As p39 expression increases, more genes in the TGFβ-EMT are involved. (B) We also evaluated the metastatic patients in TMA5 to determine if p39 expression correlates with the TGFβ-EMT signature. As p39 expression increases in stage M1 more genes in the TGFβ-EMT are involved. Statistical analysis performed was Spearman correlation. (TIF) [file pone.0207483.s005.tif]
